# Antidiabetic GLP-1 Receptor Agonists Have Neuroprotective Properties in Experimental Animal Models of Alzheimer’s Disease

**DOI:** 10.3390/ph18050614

**Published:** 2025-04-23

**Authors:** Melinda Urkon, Elek Ferencz, József Attila Szász, Monica Iudita Maria Szabo, Károly Orbán-Kis, Szabolcs Szatmári, Előd Ernő Nagy

**Affiliations:** 1 Doctoral School, George Emil Palade University of Medicine, Pharmacy, Science and Technology of Targu Mures, 540142 Targu Mures, Romania; 2 Service of Translational Medicine and Clinical Research, Emergency County Hospital Miercurea Ciuc, 530173 Miercurea Ciuc, Romania; 3 Department M3, George Emil Palade University of Medicine, Pharmacy, Science and Technology of Targu Mures, 540142 Targu Mures, Romania; 4 2nd Clinic of Neurology, Targu Mures County Emergency Clinical Hospital, 540136 Targu Mures, Romania; 5 Clinic of Diabetology, Nutrition and Metabolic Disease, Targu Mures County Emergency Clinical Hospital, 540136 Targu Mures, Romania; 6 Department of Physiology, M2, George Emil Palade University of Medicine, Pharmacy, Science and Technology of Targu Mures, 540142 Targu Mures, Romania; 7 Department of Biochemistry and Environmental Chemistry, F1, George Emil Palade University of Medicine, Pharmacy, Sciences and Technology of Targu Mures, 540142 Targu Mures, Romania; elod.nagy@umfst.ro; 8 Laboratory of Medical Analysis, Clinical County Hospital Mures, 540394 Targu Mures, Romania

**Keywords:** neurodegeneration, insulin resistance, oxidative stress, glucagon-like peptide-1 receptor agonist, animal model

## Abstract

In addition to the classically accepted pathophysiological features of Alzheimer’s disease (AD), increasing attention is paid to the role of the insulin-resistant state of the central nervous system. Glucagon-like peptide-1 receptor (GLP-1R) agonism demonstrated neuroprotective consequences by mitigating neuroinflammation and oxidative damage. The present review aims to offer a comprehensive overview of the neuroprotective properties of GLP-1R agonists (GLP-1RAs), with a particular focus on experimental animal models of AD. Ameliorated amyloid-β plaque and neurofibrillary tangle formation and deposition following exenatide, liraglutide, and lixisenatide treatment was confirmed in several models. The GLP-1RAs studied alleviated central insulin resistance, as evidenced by the decreased serine phosphorylation of insulin receptor substrate 1 (IRS-1) and restored downstream phosphoinositide 3-kinase/RAC serine/threonine–protein kinase (PI3K/Akt) signaling. Furthermore, the GLP-1RAs influenced multiple mitogen-activated protein kinases (extracellular signal-regulated kinase: ERK; c-Jun N-terminal kinase: JNK, p38) positively and suppressed glycogen synthase kinase 3 (GSK-3β) hyperactivation. A lower proportion of reactive microglia and astrocytes was associated with better neuronal preservation following their administration. Finally, restoration of cognitive functions, particularly spatial memory, was also observed for semaglutide and dulaglutide. GLP-1RAs, therefore, hold promising disease-modifying potential in the management of AD.

## 1. Introduction

Alzheimer’s disease (AD) is a commonly known neurodegenerative disorder associated with dementia in older people [1]. The disease is indeed the most frequent cause of dementia, affecting approximately 55 million people worldwide (60–70% of all dementia cases) [2], entailing a great economic impact, including healthcare, social care, and research investments [3,4]. Despite the great interest in the early diagnosis and screening of the disease, there has been no effective disease-modifying therapy (DMT) available for a long time [5]. Based on the existing human data, when memory and cognitive symptoms occur, neuronal destruction is so extensive that its remediation is no longer possible. However, short-term symptomatic relief is achievable in such cases by the approved acetylcholinesterase inhibitors (rivastigmine, galantamine, donepezil) and the NMDA receptor antagonist, memantine. In June 2021, for the first time since 2003, a new pharmacotherapeutic agent was approved by the U.S. Food and Drug Administration [6]. Despite its controversial efficacy and poor safety profile, the amyloid-β (Aβ)-targeting monoclonal antibody, aducanumab, was a major breakthrough, supporting the high demand for new therapeutic options [7]. A new pharmacotherapeutic class has thus emerged, introducing two additional drugs, lecanemab and donanemab [8,9]. Formally indicated in early symptomatic AD, these novel therapies have been shown to reduce Aβ plaque deposition and enhance Aβ clearance.

Thanks to extensive genetic, biochemical, and pathophysiological research over the past 30 years, our recent knowledge about the etiology, risk factors, development, and progression of the disease is more precise and accurate [10,11]. The currently prevailing concept of AD is a chronic, complex, Aβ and hyperphosphorylated Tau protein deposition-enhanced neuroinflammatory disease [12] that leads to progressive neurodegeneration. The synaptic loss starts in the hippocampus and extends to the frontal and temporal cortex [13], clinically manifesting in worsening cognition, memory, visuospatial, and executive abilities [14]. In accordance with the diagnostic criteria for mild cognitive impairment (MCI) or prodromal AD, the earliest cerebral changes are the decreased levels of specific Aβ isotypes in the cerebrospinal fluid (CSF) and their accumulation as extracellular aggregates (senile or neuritic plaques) in the specific brain areas [15,16]. Increased synthesis and deficient elimination could both be the reason for these alterations, depending on personal genetic and environmental risk factors [17]. The 42 amino acid fragment of Aβ is a reliable CSF biomarker of AD and negatively correlates with tissue Aβ levels [18]. The other specific hallmark of the disease is the dysfunctional modification of microtubule-associated protein Tau, which aggregates in the form of neurofibrillary tangles (NFTs), which results in neuronal cytoskeleton disturbances and synaptic loss [19]. Both amyloid and Tau pathology expand with disease progression, in which regulatory mechanisms of neuroinflammation play a substantial amplifying role. Microglia activation positively regulates the expression of β-site amyloid precursor protein cleaving enzyme (BACE1) via neuroinflammatory cytokine release and specific signal transduction cascades, increasing the activity of the amyloidogenic pathway in amyloid precursor protein (APP) metabolism [20]. Similarly, NLRP3 (nucleotide-binding oligomerization domain, leucine-rich repeat and pyrin domain containing 2) inflammasome activation regulates the activity of kinases (glycogen synthase kinase 3: GSK-3β; calcium/calmodulin-stimulated protein kinase II-α: CaMKII-α) and phosphatases (protein phosphatase 2A: PP2A) involved in Tau protein processing [21].

The amyloid cascade hypothesis served as the foundation of our understanding of Alzheimer’s disease for a considerable period. The causal role of Aβ is currently under debate, as suggested by the limited clinical success of anti-amyloid antibodies [22]. Trends toward using a single drug to affect multiple pathological processes are of growing interest. Glucagon-like peptide-1 (GLP-1) and gastric inhibitory polypeptide (GIP), two incretin hormones synthesized in the intestinal wall with a primary role in regulating insulin secretion and blood glucose levels, have been attributed with multiple neuroprotective effects in animal models, including improved long-term potentiation, synaptic plasticity, cell survival, and synapse growth and repair [23].

The present review aims to set GLP-1RAs in the context of Alzheimer’s disease and insulin resistance by presenting the GLP-1’s physiological actions and GLP-1RAs’ neuromodulating mechanisms described so far. Data concerning currently approved GLP-1RAs and incretin receptor multi-agonists are presented, highlighting behavioral, biochemical, and histological changes following treatment in preclinical animal models of AD. Possible mechanisms by which these drugs could intervene in the physiopathology of this neurodegenerative disease are also discussed.

## 2. Alzheimer’s Disease and Type 2 Diabetes Mellitus (T2DM)

It has been known for a while that patients diagnosed with type 2 diabetes mellitus (T2DM) have an increased risk of developing cognitive decline and dementia [24]. Although vascular dementia and diabetic encephalopathy also lead to neurodegeneration, in which hyperinsulinemia [25], persistent hyperglycemia [26], high levels of advanced glycation end-products (AGEs) [27,28], and associated cardiovascular risk factors [29] may be held responsible. Early epidemiological observations identified a higher incidence of AD among T2DM patients [30,31], leading to the recognition of the shared pathophysiological characteristics of the diseases [32]. Insufficient repression of glycogen synthase kinase 3 (GSK-3β) is commonly observed in both diseases [33,34]. Amylin, also known as IAPP (islet amyloid polypeptide), is a secretory product of pancreatic β-cells that regulates satiety and suppresses both insulin and glucagon secretion. Due to its chemical and physiological similarity to the Aβ peptide, it shows a tendency to aggregate in pancreatic islets of T2DM patients. Thereby, it promotes the apoptosis of pancreatic cells, just like Aβ in the brain tissue [35,36]. Many epidemiological studies have shown the relationship and important genetic linkage between T2DM and AD. The SLC2A2 gene (encoding the solute carrier family 2 member 2 protein) and the apolipoprotein E4 (apoE4) allele also share major genetic risk factors [37]. Chung et al. identified 241 candidate genes expressed in adipocytes and macrophages of AD and T2DM patients from the GSE63060 and GSE63061 datasets, representing common features of the two diseases [38]. In addition, 395 shared single nucleotide polymorphisms also suggest a common pathogenic background [39]. Both conditions are multifactorial, and among the key links identified were insulin resistance and defective insulin signaling [34,36], which are discussed in detail in Section 3.

Nonetheless, in most cases, AD develops independently of the presence of T2DM. The observation of common risk factors, genetic background, and similar pathophysiologic mechanisms inspired the term “type 3 diabetes” for AD. It also contributed to the recognition of the impact of defective insulin signaling on Aβ accumulation, Tau hyperphosphorylation, and chronic inflammation.

## 3. Insulin Resistance in Alzheimer’s Disease

Brain insulin resistance, defined as deficient insulin effect transmission due to reduced insulin receptor (IR) density or impaired cellular signaling pathway, has also been described in the context of AD progression, existing independently of peripheral insulin sensitivity [40,41]. The insulin released from pancreatic islets crosses the blood–brain barrier (BBB) in proportion to its plasma concentration via a specialized saturable transport system [42]. Cells of the central nervous system (CNS) are responsive to insulin and insulin-like growth factor 1 (IGF-1), which have mainly a neuroprotective role: promoting neuron and glial growth, cell survival, protein synthesis, and synaptic functioning [43]. The olfactory bulb, hypothalamus, hippocampus, cerebral cortex, and cerebellum have the highest density of IR. The IR-A isoform is characteristic in adult neurons, which concentrates on the post-synaptic density of post-synaptic neurons, suggesting an important role of insulin in the signaling process [44].

The IR is a tyrosine kinase type receptor whose intracellular effectors are the insulin receptor substrate (IRS) molecules. Tyrosine phosphorylation of IRS activates the insulin/p-IRS/PI3K (phosphoinositide 3-kinase)/Akt (RAC serine/threonine–protein kinase) signaling axis, which promotes neurite outgrowth and synaptogenesis via the brain-derived neurotrophic factor (BDNF) [45]. The majority of metabolic and growth-stimulating effects of insulin are mediated by the activation of PI3K and Akt, whereas Ras-MAPK (mitogen-activated protein kinase) signaling also promotes proliferation and gene transcription [46]. Translocation of the glucose transporter type 1 (GLUT1) transporter to the cell membrane of astrocytes is also a MAPK-dependent insulin effect [47]. The downstream signal transduction mechanisms are extensively reviewed by Burillo et al. [41], while Boucher et al. present the most frequently occurring phenomena that lead to insulin resistance [48].

The disruption of the intracellular signaling shifts the outputs of the phosphorylation cascade initiated through specific IRS molecules. Overactivation or increased expression of protein tyrosine phosphatase 1B’s (PTP1B) or T-cell protein tyrosine phosphatase (TCPTP) exert a negative regulatory role on neuronal insulin signaling by dephosphorylating the activated IR and its downstream effectors (IRS-2, Akt) [49,50,51]. Serine and threonine phosphorylations also hinder the biological function of the IRS [40,52]. In insulin resistance, IRS-1 is phosphorylated on Ser307 instead of activating Tyr phosphorylation. In neurons of insulin-resistant mice, 48 h long hyperinsulinemia triggered deficient Tyr608 phosphorylation of IRS-1 and Thr308/Ser473 phosphorylation of Akt. High levels of inflammatory cytokines, like interleukin 1β (IL-1β) and tumor necrosis factor-α (TNF-α), are involved in the activation of Ser/Thr kinases, thus altering the IRS-1 functioning [53,54], while interleukin 6 (IL-6) stimulates its degradation. These mediators are secreted by activated microglia and astrocytes and contribute to the reduced synthesis of BDNF [45].

Insulin resistance is a fertile background for the development of amyloid plaques in the brain. In the amyloid cascade hypothesis of AD, the amyloid-β precursor protein (APP) is cleaved through amyloidogenic processing due to the activity of the β-secretase and subsequently of the γ-secretase. The result is the formation of aggregation-prone Aβ monomers and oligomers, which form fibrils that spread extracellularly and attach as senile plaques. At the molecular level, Aβ aggregation in insulin resistance is favored directly by the deposition of IAPP seeds, to which Aβ can bind. IAPP seeds in protein aggregates were highlighted in the brains of both AD and T2DM patients [55]. Aβ binding facilitates the hyperphosphorylation of Tau, while Aβ cell membrane adhesion enhances lipid peroxidation and forms 4-hydroxinonenal, provoking perturbations of glucose and glutamic acid transport and mitochondrial damage [45].

A highly studied potential molecular culprit in the insulin-resistant state is the first enzymatic complex of mTOR (mammalian target of rapamycin). Its hyperactivation exerts neurotoxicity and enhances the Aβ deposition [56]. Preclinical studies confirmed that insulin deficiency increases the Aβ burden in 5xFAD transgenic mice by the increased expression of BACE1 and APP [57]. Besides being responsible for the cleavage of insulin, the insulin-degrading enzyme (IDE) is a means of proteolytic processing of the Aβ peptide in the CNS. Therefore, IDE polymorphism may be a common risk factor for both T2DM and AD [58].

Insulin resistance was associated with impaired glycolysis; downregulated hexokinase 2, glucose transporter type 3 (GLUT3), and glucose transporter type 4 (GLUT4) in neurons; and maintained a dephosphorylated form of the cyclin-like protein p35. The accumulation of the latter’s cleavage product, p25, is considered a driver of neuronal senescence [59]. The PI3K/Akt pathway acts as a negative regulator of GSK-3β activity. This serine/threonine kinase is a major contributor to Tau phosphorylation, with Ser396 and Ser404 being the most frequently detected sites of phosphorylation [60]. Consequently, deficient insulin signaling favors the transformation of Tau, thereby altering its physiological function [33].

## 4. Glucagon-like Peptide-1 and Its Analogues

### 4.1. Sources and Physiological Effects of GLP-1

For a long time, GLP-1 has been thought to be an endogenous peptide hormone, an incretin released by enteroendocrine cells, whose main role is the regulation of glucose homeostasis [61]. Gut-derived GLP-1 originates from the selective processing of the pro-glucagon precursor, which is 158 amino acids long and contains a sequence between 72 and 107/108 [61,62]. It was thought early on that this peptide could stimulate insulin secretion, hence the name incretin. It was discovered in the 1970s that GIP, a 42 kDA molecule that can suppress gastric motility and acid secretion, also contributes to the incretin effect [62]. Since the identification of GLP-1 depositing and releasing PPG (preproglucagon) neurons in the nucleus of the solitary tract, a peripheral-independent signaling network has been suggested [63]. Whether gut-derived GLP-1 contributes to neuronal GLP-1R activation is hard to tell, but GLP-1R distribution in the brain coincides with the axonal projections of PPG neurons, suggesting that the main stimulatory impulse is provided by the locally synthesized GLP-1 [63].

**Figure 1:**
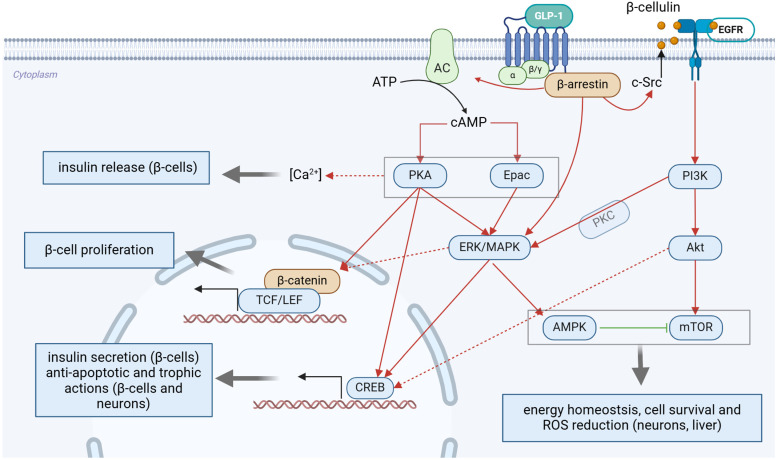
**General intracellular signaling pathways of the GLP-1R**. The G_S_ protein-coupled GLP-1 receptor, when activated by its endogen ligand, initiates three downstream signaling pathways: cAMP/PKA, PI3K/Akt, and β-arrestin-dependent ERK1/2. cAMP via PKA and Epac triggers Ca^2+^ influx and Ca^2+^ release from the endoplasmic reticulum. ERK/MAPK activation represents a crossroad in GLP-1R signaling, mediating cell-type-dependent biological functions. In pancreatic β-cells, insulin expression, proliferation, and apoptosis inhibition are transmitted by the CREB and β-catenin-TCF/LEF transcription complexes. The AMPK/mTOR balance is crucial for the energetic homeostasis of the cell, regulating protein synthesis and autophagy. Created with BioRender.com.

Gut- and brain-derived GLP-1 exerts its physiological effect by the Gα_s_ protein-coupled GLP-1R that is widely expressed in the gastrointestinal tract, cardiovascular system, and CNS [64]. GLP-1R agonism results in the cAMP (cyclic adenosine monophosphate)/PKA (protein kinase A) cascade activation. In pancreatic β-cells, PKA activation favors the closure of the ATP-sensitive potassium channel (K_ATP_ channel), while subsequent cell membrane depolarization determines voltage-gated calcium channel opening and insulin release [62]. Although exchange proteins directly activated by cAMP (Epac1 and Epac2) also play an intensifying role in rapid insulin exocytosis [65,66]. Like many other G protein-coupled receptors (GPCRs), the GLP-1 receptor has been demonstrated to induce epidermal growth factor receptor (EGFR) transactivation [67] Upon GLP-1 activation, the non-receptor tyrosine kinase, c-Src activates membrane-bound MMPs (matrix metalloproteinases) or ADAMs (disintegrin and metalloproteinase domain-containing proteins), that determine the release of β-cellulin. As a ligand for the EGFR, β-cellulin fulfills an autocrine regulatory function [68,69]. The PI3K/Akt/mTOR pathway activation [67] is thought to mediate the GLP-1’s anti-apoptotic [70,71] and neurotrophic potential [72,73,74]. Studies revealed a physical association between the scaffold protein β-arrestin and the GLP-1R, suggesting a potential role in coordinating long-lasting cellular effects [75]. It is currently hypothesized that the presence of β-arrestin serves to amplify and fine-tune certain GLP-1R signaling cascades, possibly in a G protein-independent manner [76,77]. This appears to be supported by the observation that β-arrestin elicits sustained activation of ERK1/2 and subsequent phosphorylation of the cAMP response element-binding protein (CREB) [78]. The CREB is also the main transcriptional target of PKA, which initiates the synthesis of insulin and other enzymes involved in glucose homeostasis in a fast, transient way [79]. Transcription modulation by the β-catenin-TCF/LEF (T-cell factor/lymphoid enhancer-binding factor) complex was identified as an intermediate step in β-cell survival [80]. AMPK is an energy-sensing enzyme that promotes fatty acid oxidation and glucose uptake in the liver and skeletal muscle, favoring catabolic processes [62]. Together with mTOR, AMPK regulates cellular energy metabolism and redox balance [81]. The cAMP/PKA pathway-mediated actions of GLP-1 have also been confirmed in the CNS [82,83]. CREB activation by GLP-1R triggers results in BDNF and IRS-2 expression, as well as enhancement of synaptic plasticity and memory formation [81,84]. Akt-dependent inhibition of GSK-3β resulted in β-catenin preservation. These changes are thought to be responsible for the normalization of N-acetylglucosaminyltransferase III (GnT-III) activity in the mouse brain [81]. Figure 1 displays a schematic presentation of GLP-1R signaling. There are some reports that pancreatic GLP-1Rs might be coupled with other types of G protein (G_i_ and G_q_), but their physiological significance is unclear [69,77].

The most well-known characteristic of GLP-1 is its insulinotropic mechanism. In pancreatic β cells, it favors insulin synthesis (insulin gene expression and proinsulin synthesis) and release in a glucose-dependent manner. In addition, GLP-1 inhibits glucagon secretion, thus lowering the blood glucose level. Furthermore, the systemic benefits of GLP-1 include its impact on cardiovascular homeostasis. Extensive experimental evidence is available claiming that GLP-1R signaling reduces the risk of cardiovascular events/complications. GLP-1 regulates heart rate, promotes cardiomyocyte survival, reduces end-diastolic pressure, improves stroke volume, and improves global and regional wall motion score indexes in ischemic injury and consecutive heart failure [85]. GLP-1 agonisms’ weight-lowering consequences are mediated by appetite, reward-related brain areas, and peripheral metabolic mechanisms [62,86].

### 4.2. Functional Features and Therapeutic Benefits of GLP-1RAs

Exenatide, liraglutide, lixisenatide, dulaglutide, albiglutide, and semaglutide are synthetic alternatives partially analogous to the structure of the GLP-1 polypeptide. These drugs were designed to stimulate GLP-1R receptors, thus mimicking physiologic GLP-1 actions with advanced stability and therapeutic control [87]. The identification of the naturally occurring exendin-4 and approval of exenatide was the first successful attempt for a structurally and functionally similar agent to GLP-1, whose structure is resistant to the rapid cleavage by dipeptidyl peptidase-4 (DPP-4). Simple replacements in the amino acid sequence, attachment of a fatty acid side chain (in liraglutide and semaglutide) [88], human albumin (in albiglutide), or a human immunoglobulin G4 (IgG4) Fc terminal (in dulaglutide) are strategies employed to improve the absorption and extend the half-life of GLP-1Ras; additionally, it increases lipid solubility and thus binding to serum albumin [89]. Bounded fractions of drugs form a circulating deposit that is less prone to proteolytic cleavage or renal filtration [89]. Daily or weekly sc. administration is except for semaglutide, which can be administered orally. Interestingly, despite its short mean terminal half-life, the high GLP-1R receptor affinity of lixisenatide allows once-daily dosing [90]. In the case of exenatide, a once-weekly extended-release formulation is also available, thanks to advanced microencapsulation technology [91]. Detailed structural characteristics and pharmacokinetic parameters relevant to dosing and central distribution are presented in Supplementary Table S1 and Figure S1.

The CNS penetration of the above GLP-1RAs is essential to achieve the presumed brain GLP-1R-mediated protection required in neurodegenerative diseases. Based on its satiety-enhancing and neuroprotective mechanisms, the distribution of exenatide towards the brain is now well characterized as being independent of the blood–brain barrier and more pronounced in the periventricular areas [92,93]. Intranasal administration also delivers exendine-4 to the CNS [94]. Liraglutide and lixisenatide had favorable brain/serum partition ratios with a subsequent rise in cAMP in the mouse brain over a wide dose range (25–250 nmol/kg) [95]. The arcuate nucleus and nucleus of the solitary tract have been found to be involved in the regulation of appetite and food intake by liraglutide [88,96,97,98]. These effects also support that peripherally administered liraglutide penetrates beyond the blood–brain barrier; however, the exact mechanism is still uncertain, and median eminence tanycytes may play a role in it [88,99]. At first, Salameh et al. claimed that semaglutide does not penetrate measurably behind the BBB, unlike exendin-4 and lixisenatide [100]; however, it has been demonstrated that peripherally administered semaglutide reaches biologically active concentrations in the hypothalamus [101,102]. Despite the larger molecular weight of dulaglutide and albiglutide, pharmacokinetic studies described their quick brain uptake [103,104].

GLP-1RAs are mainly new-generation antidiabetic agents with many beneficial mechanisms not only for T2DM management but also for frequently related comorbidities [105]. They reduce fasting blood glucose and glycated hemoglobin effectively [106]. Long-term use of GLP-1RAs helps to preserve and enhance the β cell function and improves peripheral insulin sensitivity [107]. Semaglutide reduces serum low-density lipoprotein (LDL) and total cholesterol concentration [106]. According to the current guidelines of the American Diabetes Association, GLP-1RAs are recommended to reduce cardiorenal risk in T2DM patients with established atherosclerotic cardiovascular disease, heart failure, and/or chronic kidney disease [108]. The reduction in major adverse cardiovascular events might be due to good glycemic control and the direct GLP-1R-mediated effect on the cardiovascular system [109]. Weight loss favoring side effects of some representatives have been observed in both T2DM and non-diabetic patients [110]. Animal experiments showed the promotion of brown adipocyte differentiation and increased energy expenditure [111]; however, in human studies, delayed gastric emptying, centrally increased satiety, and initial nausea are more evident as appetite-diminishing mechanisms [112,113,114,115]. Liraglutide and semaglutide are so efficient that their therapeutic indications have been extended for weight management in diabetic and non-diabetic patients with an initial body mass index (BMI) greater than 27 kg/m^2^ [116,117,118]. Improvement of endothelial dysfunction followed by flow-mediated dilation was described in T2DM patients [62,119].

Considering the diversity of the GLP-1R’s localization and GLP-1RAs’ widespread therapeutic actions, these drugs have a relatively good safety profile. Gastrointestinal side effects (nausea, vomiting, diarrhea) are the most frequent reasons for therapy discontinuation [106]. However, these adverse effects are dose-dependent and usually decrease over time [120]. More serious but rare complications of the therapy include acute kidney failure [121] and gallbladder and biliary diseases [122]. The early hypothesis that GLP-1RAs increase the risk of pancreatitis and pancreatic and colorectal cancer appears to be disproven, while data on the enhancement of thyroid carcinoma remain inconclusive [120].

### 4.3. Incretin-Based Multi-Agonists

The concept of incretin-based multi-agonists stems from the realization that gastric inhibitory polypeptide (GIP) may contribute to the regulation of appetite and metabolism to a greater extent than previously thought [123]. Therefore, unimolecular dual GLP-1/GIP receptor agonists have been developed and clinically tested for the management of T2DM and obesity [124,125]. Tirzepatide is the only dual agonist approved by the U.S. Food and Drug Administration (FDA) and European Medicines Agency (EMA) so far. It proved to be superior to semaglutide in ameliorating T2DM and obesity-related outcomes [126]. Similar trends are revealed by the meta-analysis of Yao et al. [106]. Based on this success, other combinations of simultaneous G protein-coupled incretin receptor activation have been proposed [127,128,129]. Coactivation of GLP-1, GIP, glucagon, amylin, and peptide YY receptors, besides metabolic effects, could deliver cardiovascular, renal, and hepatic benefits in the future [128,129]. High expectations are set for the GLP-1/GIP/glucagon receptor triagonist, retatrutide, that is currently evaluated in phase 3 clinical trials. It is promising not only for T2DM and weight reduction but also for metabolic dysfunction-associated steatotic liver disease (MASLD) [128].

The GIP receptor is also widely expressed in the CNS, especially in appetite and energy homeostasis-controlling regions in the hindbrain and hypothalamus [130]. The GIP receptor takes part from the same receptor family, and as such, cAMP/PKA-driven CREB and ERK1/2 activation are the main facilitators of most effects. However, the activation of the PI3K/Akt pathway differentiates between the two receptors, as it is not specific to the GIP receptor [131,132]. Tissue-specific effects of pharmacological co-agonism on GLP-1, GIP, and glucagon receptors are summarized by Nogueiras et al. [129]. To date, the majority of CNS effects-focusing studies have discussed the GIP agonism’s mechanisms of food intake and appetite control [133,134], and there is less known regarding neuroprotection.

In pharmacokinetic studies, some incretin receptor co-agonists have similar or better brain penetration than GLP-1RAs [100,135]. Hölscher et al. developed a series of experimental dual GLP-1/GIP receptor agonists with peptide structure to test in neurodegenerative disease models [100,136,137]. Supplementary Table S1 also contains comparative information on the structural and pharmacokinetic characteristics of tirzepatide and retatrutide.

Since single GIP receptor agonism also demonstrated neuroprotective properties [138,139] and incretin-based multi-agonists have been shown to inhibit neurodegeneration in cellular models [140], these newly developed agents are suitable candidates as AD therapies.

## 5. GLP-1R Activation in the Context of Neuroprotection

### 5.1. GLP-1 and GLP-1RAs Exert Specific Neuroprotective Effects

Neural circuits are exposed to the biochemical modulatory effects of pro-inflammatory and anti-inflammatory astrocytes and glial cells. Heterogeneous populations of astrocytes and microglia with many transitional types from A1/M1 to A2/M2 are responsible for neurotoxicity and repair [141]. Microglial activation and switch to M1, along with astrogliosis, propagates through the release of reactive oxygen species (ROS) and pro-inflammatory cytokines such as IL-1, IL-6, IL-17, and prostaglandin E2 (PGE2), and can directly damage neighboring neurons via complement activation [140]. A strong link between insulin resistance, neuroinflammation, and subsequent neurodegeneration has been demonstrated in the transgenic mouse model of APP/PS1. Activated astrocytes produce higher levels of proinflammatory cytokines, IL-1β, and interferon-γ (IFN-γ) and show phosphorylation of IRS-1 at Ser616, characteristic of insulin resistance [140]. GLP-1R triggering silences typical inflammatory signaling axes, such as JAK/STAT3 (Janus kinase/signal transducers and activators of transcription), and suppresses nuclear factor κB (NF-kB), an important transcription factor of pro-inflammatory cytokines via Akt/CREB (cAMP response element-binding protein) in macrophage/microglia cells [142].

Diverse neuroprotective, antiapoptotic, insulinotropic, anti-oxidative, and anti-inflammatory effects of GLP-1 have been described in the CNS [140]. Central GLP-1 signaling proved to exert multiple neuroprotective effects, including neurite outgrowth and protection against glutamate excitotoxicity, apoptosis, and stimulation of tyrosine hydroxylase synthesis, which enhances dopaminergic neurotransmission [62]. Neurons, astrocytes, and glial cells all express GLP-1R [140]. In rodents, GLP-1R is abundantly expressed in the hippocampus, and its activation improves learning and memory [143,144]. GLP-1R knockout mice showed learning deficits that could be rescued by inserting the Glp-1r gene into the hippocampus [143]. Neurons in the hippocampus of the rat treated with GLP-1 or its analog, exendin-4, are more resistant to the excitotoxicity of glutamate, whereas GLP-1R overexpression increases the viability and proliferative capacity of human neuroblastoma cells [140]. GLP-1 and the receptor agonist exendin-4 induce differentiation and neurite outgrowth in rat pheochromocytoma cells. GLP-1 and exendin-4 confer protection for hippocampal neurons against apoptosis induced by glutamate [145]. Pharmacological silencing of PI3K and ERK suspends neurite outgrowth in rat PC12 cells, but this effect is independent of PKA since it is not achieved with its upstream inhibition [62]. Both GLP-1 and exendin-4 reduce the depletion of cholinergic bodies in hippocampal neurons induced by the neurotoxic non-proteinogenic amino acid, ibotenic acid [145]. These effects could be beneficial in reducing the neuroinflammatory microenvironment created in an insulin-resistant background.

### 5.2. GLP-1RAs Down-Regulate Chronic Oxidative Stress of the CNS

The so-called redox priming and low levels of H_2_O_2_ are net positive regulators of IR activation by the inhibition of PTP1B phosphatase [44]. In contrast, in neurons, high levels of ROS in some circumstances promote the formation of NLRP3 inflammasome or alter the expression of methyltransferases and epigenetic regulation, determining apoptosis [146]. Excessive oxidative stress may be a key trigger in the long path of neurodegenerative deterioration [147]. High insulin sensitivity in healthy cells is associated with low levels of ROS and low redox signaling [34]. Conversely, metabolic dysregulation and inflammatory signals in neurodegenerative diseases both trigger increased production of reactive oxygen and nitrogen species (ROS and RNS). Neurons with high oxygen demand, high-level mitochondrial respiration, high levels of structural lipid substrates, and low defensive antioxidant enzymes are especially susceptible to oxidative stress [146]. Excess glucose in T2DM generates Amadori products, which accumulate on various proteins and excite the receptors for advanced glycation end-products (RAGEs). In addition, enediol radicals formed from glucose excess synthesize superoxide, hydroxyl, and peroxynitrite radicals.

Several publications support the specific importance of oxidative damage in AD. Carbohydrate metabolism defects and mitochondrial ROS overproduction induced by Aβ and NFT deposition lead to a decline in synaptic activities on the facilitating background of suppressed glutathione levels, Zn^2+^/Cu^2+^ superoxide dismutase (SOD), and catalase enzyme activities in the frontal and temporal cortex [147,148]. In a human study of a cohort of participants in the Chinese Longitudinal Healthy Longevity Survey (CLHLS), higher serum SOD activity was associated with poor scores on the Chinese version of the Mini-Mental State Examination (MMSE) [149].

The extracellular accumulation of Aβ itself is a major source of free radicals via metal ion-catalyzed oxidative cleavage of APP. Aβ induces peroxide and hydroxyl production along with lipid peroxides, causing hypothalamic neurotoxicity through activation of NADPH oxidase [150]. By-products like 4-hydroxy-2-nonenal may cause excessive Tau phosphorylation and loss of hippocampal neurons [147].

Epac activation by GLP-1 receptor triggering is required for the synthesis of the antioxidant enzymes catalase, glutathione peroxidase 1, and manganese-dependent superoxide dismutase and for the suppression of H_2_O_2_ production [140,151,152]. GLP-1 and exendin-4 were shown to significantly reduce the amount of endogenous H_2_O_2_ in SH-SY5Y neuroblastoma cells [153]. GLP-1 also ameliorated mitochondrial dysfunction through cAMP/PKA pathway activation in Aβ-treated astrocytes [154].

GLP-1 signaling may also interfere with oxidative stress at the level of antioxidant enzyme gene expression, as it regulates the nuclear erythroid-2-like factor-2/antioxidant response element (Nrf2/ARE) pathway via PI3K and Akt activation [155]. Nrf2, together with Kelch-like ECH-associated protein 1 (Keap), controls a number of antioxidants such as superoxide dismutase, catalase, glutathione peroxidases, thioredoxin, thioredoxin reductase, sulfiredoxin, nicotinamide adenine dinucleotide phosphate (NADPH) oxidoreductase-1, heme oxygenase-1, glutathione reductase, glutaredoxin, glutathione S-transferase, uridine diphosphate (UDP)-glucuronyl transferase [156]. Liraglutide, a GLP-1RA, was shown to reduce not only infarct size in experimental cerebral ischemia induced by middle cerebral artery occlusion but also the level of lipid peroxidation by suppressing the upregulation of superoxide dismutase and glutathione peroxidase [157]. In another experimental study, liraglutide decreased ROS production after oxygen/glucose deprivation in primary rat neurons and exerted neuroprotection by reducing infarct size via activation of PI3K/Akt and MAPK pathways [158]. In a traumatic brain injury model, this GLP-1RA rescued cells from H_2_O_2_-induced cell death [153]. According to several preclinical and human studies, sitagliptin, a DPP-4 inhibitor that increases the levels of endogenous GLP-1; simultaneously suppresses the pro-inflammatory factor and cytokine production of NF-κB, IL-6, IL-17, IFN-γ, and TNF-α, reactive oxygen species; and upregulates the anti-inflammatory factors, IL-10 and transforming growth factor β (TGFβ), the anti-oxidant glutathione, and M2-type macrophage/microglia [159]. Thus, the reduction in ROS overproduction in AD may not be a specific and solitary therapeutic target but rather part of the complex metabolic benefits of GLP-1RA applications.

## 6. GLP-1RAs in Experimental Models of AD

The current chapter presents the existing preclinical data regarding the possible benefits of these new-generation antidiabetic agents in AD with the aim of a better interpretation of the individual results. The PubMed database was searched for references up to 31 December 2024 using the following criteria: [drug name] AND (animal*) AND (Alzheimer*). Only data relevant to AD, neuroinflammation, neurodegeneration, and central insulin resistance were summarized in the tables. Studies investigating the central effects of GLP-1RAs in animal models of T2DM were not included in the current review, as its main purpose was to evaluate a potential neuroprotective effect of GLP-1RAs in the AD characteristic neurodegeneration, and not in the diabetes-associated cognitive impairment.

### 6.1. Exenatide

No animal model is perfect in all respects; while genetic animal models, such as APP/PS1 or 3xTg-AD, are perfect in modeling a discrete pathomechanism, they fail to mimic all the typical functional and structural modifications at the same time. Interventional animal models involve the creation of chemical or physical lesions in a particular brain region that symptomatically more closely resemble late-onset AD yet result in an advanced disease state with a diminished probability of favorable pharmacological intervention [160]. As presented in Table 1, exenatide was tested in five different models of transgenic mice: APP/PS1, 3xTg-AD, PS1-KI, 5xFAD, and Tg2576, all of which are well-established classical AD murine models that focus on Aβ accumulation, associated or not with Tau hyperphosphorylation [161]. Some of these studies concluded that exenatide (daily doses of 100, 200 µg/kg body weight) had a positive influence on memory, tested exclusively with the Morris Water Maze (MWM) [162,163,164,165]. In contrast to the above studies, Bomba et al., who also evaluated the influence of the high-fat diet (HFD) in 3xTg-AD mice, concluded that the effects of exenatide (500 µg/kg) were diet-dependent [166]. An earlier study by the same author had similar conclusions despite a longer treatment period (9 months). Exenatide did not improve cognitive performance or histological hallmarks of AD in 3xTg-AD mice [167]. The behavioral, biochemical, and histological consequences of exenatide treatment were also tested in the three most commonly used interventional rodent models: Aβ-, lipopolysaccharide (LPS)- or streptozotocin (STZ)-induced neuroinflammation. Two studies applied complex AD and T2DM models with additional ip. administration of STZ [168,169]. While central administration of STZ triggers sporadic AD-like pathology [170], peripheral dosing induces pancreatic β-cell-specific toxicity [171]. The MWM is the most widely used behavioral test for assessing spatial learning and memory. The animal’s endeavor is to evade the aqueous environment and seek refuge by the aim of available proximal land distal visual cues. Since it can incorporate control for perceptual and motivational factors, it is a reliable and robust method suitable for rodents of different ages and activity levels [172]. The Barnes Maze test is a swim-free alternative for the evaluation of hippocampus-dependent spatial memory. The escape-seeking principle is the same; however, it is more suitable for rodents with impaired mobility or pronounced depressive-like behavior [173].

Some studies still try to explore the cause-and-effect relationships of Aβ accumulation. The study conducted by Ohtake et al. brought to our attention that following five doses of exenatide disintegrin and metalloproteinase domain-containing protein 10 (ADAM10), activity was increased, while BACE1 remained unchanged, favoring the non-amyloidogenic processing of APP [174]. Nevertheless, the impact on the soluble Aβ fraction and Aβ plaque load remains inconclusive, with a similar number of positive and negative outcomes (Table 1). The complex experimental model designed by Huang et al. (ip. STZ and intrahippocampal LPS) evidenced that exendin-4 could be useful not just in preventing diabetes mellitus-mediated neurodegeneration but also in alleviating neuroinflammation itself by decreasing microglia and astrocyte activation [175]. Similarly, Li et al. and King et al. observed reduced hippocampal amyloid pathology and neuroprotection in complex AD and T2DM models [168,169]. Reduction in TNF-α was associated with better choline acetyltransferase (ChAT) activity and better preservation of hippocampal neurons [176]. As stated in the comprehensive study of Garabadu et al., a similar influence on cholinergic neurotransmission was observed [177]. Exenatide reduced pro-inflammatory cytokines, TNF-α, and IL-1β in the cerebral cortex of 5xFAD mice. This phenomenon has been linked to a reduction in NLRP2 inflammasome and astrocyte activation [165]. In a consistent finding, diminished caspase 3 activity was documented in the STZ-induced Alzheimer-like disease model, with neuroprotection being confirmed by better learning performance [178]. The data regarding exenatide’s effects on neuroinflammation are currently expanding, while several studies suggest other neuroprotective mechanisms. A single dose of intracerebroventricular (icv.) or intranasal exenatide had antiapoptotic properties, protecting from Aβ-induced neurotoxicity, confirmed by the MWM in two different studies [94,179]. According to Wang et al., a single intrahippocampal injection of exendin-4 protects against Aβ_1-42_-induced impairment of calcium homeostasis and long-term potentiation (LTP) [180]. These findings resemble two other studies that described enhanced neurotrophic activity [166] and synaptic integrity [163].

Lastly, effects on IR and GLP-1R signaling are crucial to understanding the eventual neuroprotective properties of GLP-1RAs. Exenatide improved the deficient IR signaling, which is reflected in the reduced serine phosphorylation of IRS-1 [164] and regulation of the downstream Akt/GSK-3β/β-catenin pathway [162]. Our current knowledge suggests that GSK-3β hyperactivity represents a pivotal node in the pathomechanism of AD, which connects the majority of the previously discussed mechanisms [181]. Following exendin-4, a decrease in GSK-3β activity was measured, accompanied by a reduction in Tau phosphorylation [182]. This outcome seems to confirm the theory that GSK-3β is directly involved in the phosphorylation of Tau. Interestingly, intranasal coadministration with insulin has been shown to reduce the gene expression of some IR cascade molecules (IRS1, INSR, AKT1, AKT3, GSK-3β) without significant changes in monotherapy [183]. Furthermore, it did increase IR immunoreactivity in the CA1 region of wild-type animals but not in icv. STZ-treated animals [178].

The studies have not revealed where GLP-1R signaling meets the previously mentioned effector system to date. Elevated expression of GLP-1R in microglia and astrocytes, but not neurons, was observed in 5xFAD and 3xTg-AD, evidencing the anti-inflammatory potential of exenatide [184]. In addition, enhanced BDNF signaling or TNFα reduction could also contribute to Akt/GSK-3β regulation [185].

**Table 1:** Studies assessing exenatide in animal models of Alzheimer’s disease.

Study	Animal Model	Route of Administration and Dosage	Results		
			Behavior	Biochemistry	Histology
[168]	3xTg-AD mice ± ip. STZ	micro-osmotic pump at 3.5 pM/kg/min rate (sc.), 16 weeks	n.d.	↓ APP, soluble Aβ oligomer in the hippocampus no diff. in total Tau	↓ Aβ plaque load in the hippocampus
[164]	APP/PS1 mice	25 nmol/kg ip., daily for 3 weeks	MWM: ↓ escape latency and distance moved, ↑ time in target quadrant;	↓ Ser363-, Ser 312-p-IRS-1, and p-JNK in the hippocampus ↓ soluble Aβ	↓ Aβ plaque load in the cerebral cortex
[175]	C57BL/6J mice, intrahippocampal LPS, and ip. STZ	10 µg/kg ip., daily for 28 days	MWM: ↓ escape latency and ↑ time in target quadrant;	↓ COX1, COX2, CD45, NF-κB in the hippocampus	↓ Iba-1+ microglia and GFAP+ astrocyte in the hippocampus; ↑ Tyr-hydroxylase (in locus coeruleus) and serotonin immunoreactivity (in raphe nucleus)
[182]	Wistar rats, icv. STZ	10 μg/kg sc., twice a day, 14 days	MWM: ↓ escape latency and distance moved, ↑ time in target quadrant	↓ Ser396-, Thr181-p-Tau ↑ Ser9-p-GSK-3β, ↓ Tyr216-p- GSK-3β and total GSK-3β	↑ density of CA1 normal neurons
[167]	PS1-KI and 3xTg-AD mice	500 µg/kg ip., daily, 5 days a week, 9 months	MWM: no statistically significant difference (both strains)	no modification of COX (both strains), ↑ forward LDH activity (PS1-KI) in brain homogenate	no reduction in hippocampal Aβ load and hyperphosphorylated-Tau (3xTg-AD)
[174]	CD1 mice, icv. Aβ_1-42_	0.2 mg/kg sc., five doses given at 3-h intervals;	n.d.	↑ the plasma membrane GluR1 and ADAM10 in the neocortex	n.d.
[176]	Sprague Dawley rats, icv. STZ	20 µg/kg ip., daily for 14 days	PAL: ↑ latency	↓ TNF-α, ↑ ChAT	↑ neuron count in CA1 and CA3 regions
[94]	C57BL/6 mice, intrahippocampal Aβ_31-35_	single dose of 0.5 nmol intranasal or 0.05 nmol intrahippocampal drug, 30 min prior to Aβ_31-35_	MWM: ↓ escape latency and ↑ time in target quadrant; ↑ locomotor activity	n.d.	n.d.
[179]	Sprague Dawley rats, icv. Aβ_1-42_	single dose of 0.02 nmol, 0.2 nmol, 2 nmol drug, 15 min before the Aβ_1-42_	MWM: ↓ escape latency and distance moved, ↑ time in target quadrant (0.2 and 2 nmol)	↑ Bcl2 mRNA, ↓ Bax mRNA and caspase-3 in the hippocampus (0.2 and 2 nmol)	n.d.
[162]	APP/PS1 mice	25 nmol/kg sc., twice a day, 4 weeks	MWM: ↓ escape latency, ↑ time in target quadrant and number of target crossings	↓ GnT-III, GlcNAc and ↑ Ser473-p-Akt, Ser9-p-GSK-3β, β-catenin in the hippocampus	↓ GnT-III expression in cortex and hippocampus
[163]	5xFAD mice	100 µg/kg sc., twice a day, 16 weeks	MWM: ↓ escape latency, ↑ number of target crossings	↑ PSD95 and SYN and normalized mitochondrial dynamics in the hippocampus	↓ Aβ plaque number, ↑ specific surface area of mitochondria in the CA1 region
[166]	3xTg-AD mice ± 6 months-long high fat diet (HFD)	500 µg/kg ip. for five days per week, 3 months;	No difference in MWM and ORT	↑ BDNF level and improved it’s signaling (HFD and non-HFD) ↑ Ser1101-p-IRS-1 (HFD), ↓ NF-κB and PPARα/γ (HFD)	No difference in Aβ and p-Tau load in the CA1 region
[183]	Tg2576 mice	0.075 μg intranasal, 6 days a week, 8 months ± 0.43 × 10^−3^ IU insulin	MWM: ↓ escape latency (monotherapy)	no difference in hippocampal Aβ_1-42_ ↓ IRS1, INSR, AKT1, AKT3, CTNNB1, GSK-3β, IGF-1R mRNA in the hippocampus (combination only)	n.d.
[177]	Wistar rats, icv. Aβ_1-42_	5 μg/kg ip., daily for 14 days	MWM: ↓ escape latency, ↑ time/distance in target quadrant Y-maze: ↑ spontaneous alternation behavior	↑ ACh, ChAT activity, ↓ Aβ, AChE activity in hippocampus and prefrontal cortex	n.d.
[169]	APP_swe_/Tau mice ± ip. STZ	10 μg/kg sc., twice a day, 6 weeks	Barnes circular maze: no diff. in escape latency and error	↓ soluble Aβ oligomer, no diff. in Thr231-p-Tau	↑ NeuN+ neurons in the hippocampus (APP/Tau+STZ only)
[165]	5xFAD mice	100 μg/kg sc., twice a day, 16 weeks	MWM: ↓ escape latency, ↑ number of platform crossings	↓ TNFα, IL-1β, GFAP and NLRP2 expression in the cortex	↓ Aβ_1-42_ load, GFAP+ astrocyte and NLRP2 in the cortex ↑ NeuN+ neurons in the cortex
[178]	Wistar rats, icv. STZ	10 μg/kg ip., daily for 21 days	Y-maze: ↑ frequency and time in the novel arm ORT and ODT: ↑ recognition index	n.d.	↑ NeuN+ neurons and ↓ cleaved caspase-3 immunoreactivity in the CA1 and CA3 regions and dentate gyrus

The table presents statistically significant results (no trends of increase/decrease), the arrows indicating the direction of change/difference following drug administration compared to placebo-treated animals with AD. Akt: RAC-alpha/gamma serine/threonine–protein kinase; Aβ: amyloid-β; BACE1: β-site amyloid precursor protein cleaving enzyme; BDNF: brain-derived neurotrophic factor; CA1: *Cornu Ammonis* 1; CREB: cAMP response element-binding protein; GFAP: glial fibrillary acidic protein; GLP-1R: glucagon-like peptide-1 receptor; GLUT1: glucose transporter type 1; GSK-3β: glycogen synthase kinase-3; G6PDH: glucose-6-phosphate dehydrogenase; HK: hexokinase; Iba-1: ionized calcium-binding adaptor molecule 1; IL-1β: interleukin 1β; IRS-1: insulin receptor substrate 1; MWM: Morris Water Maze; n.d.: no data; NOR: Novel Object Recognition test; ORT: Object Recognition Task; PFK: phosphofructokinase; PINK1: PTEN-induced kinase 1; PI3K: phosphoinositide 3-kinase; PSD95: postsynaptic density protein 95; Ser: serine; STZ: streptozotocin; SYN/SYP: synaptophysin; TEM: transmission electron microscopy; Thr: threonine; TNF-α: tumor necrosis factor-α.

### 6.2. Liraglutide

A substantial body of research has been conducted on the effects of liraglutide in animal models of AD, as shown in Table 2. The majority of the studies employ the APP/PS1 transgenic mouse strain with the Swedish mutation. This strain exhibits progressive Aβ plaque deposition, accompanied by adjacent gliosis and synapse loss, but no NFTs [186]. The senescence-accelerated mouse-prone 8 (SAMP8) is a naturally occurring strain that displays age-associated Aβ formation (no plaques) and behavioral impairments; hence, it is not highly specific for AD [187]. Liraglutide had a positive impact on plaque-independent cognitive deficit and hippocampal neuron preservation in SAMP8 mice [188]. A phenotypically similar model is the PLB4 transgenic mouse strain, which is characterized by the additional expression of human BACE1 [189]. It is noteworthy that these models (SAMP8 and PLB4) reproduce memory impairment without plaque deposition or Tau phosphorylation, thereby questioning their causal role. In addition to the classic Aβ- and STZ-induced neurodegeneration, a homocysteine-induced AD-like pathology design was also used to test liraglutide [190]. It has been shown that continuous hyperhomocysteinemia triggers central Aβ and Tau pathology, as well as cognitive decline [191,192]. The study by Zhang et al. showed that concomitant treatment with liraglutide proved beneficial in all these aspects [190]. Liraglutide was also tested in two complex animal models. Paladugu et al. investigated the effect of liraglutide in 5xFAD transgenic and icv. STZ treated animals in parallel and in combination, highlighting important differences [193]. Wild-type animals receiving STZ were less responsive to treatment in most outcomes compared to 5xFAD mice, while in combined 5xFAD-STZ animals, liraglutide could achieve minimal beneficial influence [193]. In the study by Carranza-Naval et al., APP/PS1xdb/db crossbred mice were used to assess whether the effects of liraglutide are dependent on the coexistence of T2DM [194]. Liraglutide demonstrated restorative properties in both diseases, although these were not necessarily interdependent. Lastly, it is noteworthy that liraglutide has also been tested on a small number of macaques exhibiting AD-like pathology induced by icv. administration of Aβ oligomers [195]. This non-human primate model has proven to serve as a reliable representation of various pathological features of AD, including Tau hyperphosphorylation and tangle formation, synapse loss, astrocytic/microglial activation, and impairment of IR signaling; therefore, it is a valuable asset in the research of AD therapies [196].

In experimental models, the MWM continues to be the most widely used behavioral test for memory impairment associated with AD. However, animal studies of liraglutide are more often completed with the Novel Object Recognition (NOR) test (Table 2). This test is based on the rodents’ propensity to investigate the previously not encountered object [197]. According to the study conducted by Baker et al., the hippocampus is only involved in object recognition when object location and recency concepts are applied [198]. In this regard, the object location memory (OLM) [199] and new object discrimination (NOD) [194,200] tests are valuable alternatives, being more sensitive to hippocampal neurodegeneration. This does not invalidate the use of the NOR test as experimental models of AD do not present solely hippocampus-specific impairment but also neurodegeneration of the frontal cortex. The Y- and T-maze are relatively simple tests used for spatial working memory and reference memory acquisition [201,202]. Different protocols may employ spontaneous or rewarded alternation, thus providing slightly different conclusions. Contextual fear conditioning evaluates whether the animals can associate a certain environment (context) with an aversive stimulus [203]. Upon encountering the previously learned context, animals respond with freezing behavior, indicative of the functionality of the amygdala, hippocampus, and frontal cortex [204]. Of the behavioral tests listed, NOR causes the least emotional distress, while the others involve different stimuli, such as contact with water, food deprivation, or mild pain.

Besides AD-specific outcomes, some studies also assess peripheral glucose homeostasis (glycemia, HbA1c, plasma insulin) and changes in body weight. Holubova et al. detected a significant reduction in blood glucose [205]; however, based on GLP-1RAs’ pharmacologic mechanism, this effect only occurs when hyperglycemia is present [206]. This is supported by the fact that liraglutide did not elevate plasma insulin concentrations in non-diabetic mice [207,208,209,210]. The central effects of the GLP-1RAs in AD are, therefore, independent of the presence of insulin. The same study documented a 7% reduction in body weight [205], while other publications found that body weight has been maintained [188,194,206,207,208,209,211] following long-term liraglutide administration. These studies support the findings that liraglutide reduces body weight only in overweight and obese animals.

The effects of liraglutide were evaluated on many aspects of Aβ deposition and APP processing (Table 2). Reduction in soluble Aβ and APP was measured in APP/PS1 [212,213,214], 5xFAD [193], and 3xTg-AD mice [209] with a consequent decrease in density of Aβ plaques in the hippocampus and/or cortex [194,205,210,212,213,214,215,216,217,218,219]. In contrast, long-term liraglutide administration had no effect on Aβ deposition in two transgenic mouse models of central amyloidosis [220]. In two cases, a reduction in APP was also detected [212,213], as well as reduced phosphorylation of APP at the position of 668 [190], which is known to favor the amyloidogenic pathway [191]. Similarly to the effect observed at exenatide [174], liraglutide also enhanced the α-secretase activity, accompanied by BACE1 reduction, suggesting a beneficial shift in APP processing [190]. In addition to the previously mentioned reduction in Aβ synthesis, an enhanced degradation can be an effective strategy against amyloid pathology. It has been shown that patients diagnosed with mild cognitive impairment or AD present low levels of IDE in their brains [221]. This β-sheet structure-specific proteolytic enzyme actively participates in the clearance of neurotoxic oligomer forming Aβ [222]. Following liraglutide treatment, increased IDE levels were detected in two different animal studies [193,212]. According to a novel approach, the glymphatic system has been suggested to participate in Aβ clearance [223]. Sasaki et al. observed aquaporine-4 (AQP4) relocalization to the perivascular astrocyte plasma membrane to enhance Aβ elimination via the CSF [210].

A substantial consensus on Tau protein modification can be observed in the studies discussed (Table 2). Rodents treated with liraglutide exhibited a reduced degree of Tau modification at many different sites susceptible to phosphorylation [190,194,206,207,211,224]. This trend was also confirmed in non-human primates (NHPs) [195]. Dysregulation of PP2A has been suggested to be a major culprit in Tau phosphorylation and microtubule destabilization; however, given the vast diversity of this serine/threonine phosphatase family and its versatile regulation, it is challenging to establish direct causality [225].

The activation of the cAMP/PKA pathway was measurable following seven days- and eight weeks-long administration of 25 nmol/kg liraglutide, thereby suggesting GLP-1R activation in the CNS [154,195]. As demonstrated in the study by Zhang et al. liraglutide protects against the hyperhomocysteinemia-induced decline of GLP-1R protein in the hippocampus [190]. Similarly, Qi et al. and Gao et al. also detected higher levels of GLP-1R protein in the hippocampus of the treated group [207,214]. The impact of the treatment on the brain’s IR expression and activation remains ambiguous. Some studies found no difference in the gene expression of the two subtypes of IR (IR-A and IR-B) [194,212]. However, Batista et al. detected increased expression of the α-subunit of IR in the hippocampus of mice and IR immunolabeling in the frontal cortex of NHPs [195]. Evidence suggests that by exposure to Aβ, a redistribution of IRs to the cell body from dendrites can be observed [226]. Furthermore, liraglutide elicited an increase in IR phosphorylation within the cortex, albeit not in the hippocampus of 5xFAD and 5xFAD-STZ mice [193]. Dysregulating Ser616 and Ser312 phosphorylation of IRS-1 was confirmed in two distinct models as markers indicative of an insulin-resistant state [190,216]. In both cases, liraglutide treatment proved to have restorative properties on IR signaling [190,216]. The relationship between Aβ pathology and central insulin resistance is indirect, as evidenced by the identification of increased neuronal Ser616-p-IRS-1 not only in the immediate proximity of Aβ plaques but also in cortical regions not affected by amyloid pathology [216]. In accordance with the aforementioned rationale, improvement in PI3K/Akt pathway activity could also be observed in APP/PS1 and 5xFAD mice [214,227], as well as negative regulation of GSK-3β [193,207,214]. ERK and JNK (c-Jun N-terminal kinase), which are members of the MAPK superfamily, function as signal transduction molecules that link deficient insulin signaling and neuroinflammation [228]. The role of MAPK kinases is complex, but they are responsible for coordinating a variety of transcriptional processes. Nonetheless, preserved phosphorylation of ERK and JNK suggests a potential therapeutic benefit of liraglutide [206,211,219].

Several studies employing different transgenic and interventional animal models have described an actual neuroprotective effect of liraglutide, manifesting as preservation of neuronal density and synaptic integrity in the cortex and hippocampus [154,188,194,206,211,212,213,214,215,217,227,229]. In most cases, this was associated with reduced microglia and astrocyte activation [193,210,216,218,219]. Interestingly, Holubova et al. showed that following liraglutide treatment, colocalization of cortical Aβ plaques with reactive astrocytes was ameliorated in the cortex but not in the hippocampus [205]. According to Zheng et al., liraglutide mitigates neuronal oxidative stress by promoting the aerobic glycolysis of astrocytes [227]. Recent studies demonstrated that liraglutide may be beneficial in mitigating AD-related oxidative stress by reducing reactive carbonyl and nitrite levels and increasing glucose-6-phosphate dehydrogenase (G6PDH) expression [154,209]. Enhancement in LTP was documented following chronic ip. administration of 2,5 and 25 nmol/kg liraglutide [212,230]. These findings are consistent with the current understanding of AD-related neuroinflammation [231]. However, there is a lack of evidence regarding the influence of liraglutide on inflammatory biomarkers (e.g., IL-1β, TNF-α, IL-6) and regulatory mechanisms (e.g., NF-kB, p38 MAPK, mTOR). Mengr et al. detected lower brain NF-κB activity following four months of 0.2 mg/kg/day liraglutide administration in 3xTg-AD mice [219], while Duarte et al. found a reduction in IL-10 and plasma IL-1β, although not statistically significant [209].

**Table 2:** Studies assessing liraglutide in animal models of Alzheimer’s disease.

Study	Animal Model	Route of Administration and Dosage	Results		
			Behavior	Biochemistry	Histology
[194]	APP/PS1xdb/db crossbreed mice	500 μg/kg sc., daily for 20 weeks	MWM: ↓ escape latency, ↑ time in target quadrant NOD: ↑ “what” and “where” paradigms Rotarod: no difference in time and speed	no difference in soluble and insoluble Aβ (cortex and hippocampus) ↓ p-Tau (cortex) no difference in IR-A, IR-B, IGF-1R mRNA	↑ brain weight and cortex size ↑ neuronal density in cortex and hippocampus ↓ Aβ plaque burden in cortex (APP/PS1), no difference in APP/PS1xdb/db animals ↓ Iba-1 + microglia in cortex
[227]	5xFAD mice	25 nmol/kg sc., daily for 8 weeks	MWM: ↓ escape latency, ↑ number of target crossings	↑ PSD95 and SYN in cortex ↑ glycolysis, ATP production, p-PI3K, p-Akt, ↓ ROS	↑ neuronal density in cortex
[190]	Sprague-Dawley rats, intravenous homocysteine	150 (L), 300 (M) and 450 μg/kg (H), twice a day, 2 weeks	MWM: ↓ escape latency, ↑ distance in target quadrant (L, M)	↓ plasma homocysteine (L, M) ↓ Thr231-, Ser404-, Thr205-p-Tau, PSEN1 (L, M), Aβ, Ser668-p-APP, BACE1, demethylated and p-PP2A (all), ↑ ADAM10 (L, M) ↓ Ser312-p-IRS-1, ↑ GLP-1R (all)	↓ Thr231-p-Tau
[193]	5xFAD and wild-type mice, icv. STZ	25 nmol/kg ip., daily for 30 days	OF, NOR, PAL: no statistically significant difference	↓ Aβ only in 5xFAD-nonSTZ mice ↑ IDE (both), ↑ p-IR in cortex, ↑ p-GSK-3β in hippocampus and cortex (5xFAD, 5xFAD-STZ)	no diff. in the number of degenerating neurons ↓ GFAP+ astrocytes and Iba-1 + microglia (cortex, CA1, CA3) (both)
[216]	APP_swe_/PS1_dE9_ mice	25 nmol/kg ip., daily for 8 weeks	n.d.	n.d.	↓ Aβ plaque number, IRβ expression, S616-p-IRS-1, GFAP+ astrocytes and Iba-1+ microglia in frontal cortex
[212]	APP_swe_/PS1_dE9_ mice	25 nmol/kg ip., daily for 8 weeks	NOR: ↑ recognition index MWM: ↑ time and distance in target quadrant	↓ soluble Aβ oligomers and APP no difference in IR, BDNF, Ngfr mRNA ↑ IDE mRNA	↓ Aβ plaque load, ↓ Iba-1 + microglia, ↑ Doublecortin+ neurons, ↑ synapse number in the hippocampus
[213]	APP_swe_/PS1_dE9_ mice	25 nmol/kg ip., daily for 8 weeks	NOR: ↑ recognition index MWM: ↓ escape latency, ↑ time and distance in target quadrant	↓ soluble Aβ oligomers and APP	↓ Aβ plaque load, ↓ Iba-1 + microglia in the cortex ↑ Doublecortin+ neurons ↑ synapse number in the hippocampus
[215]	APP_swe_/PS1_dE9_ mice	25 nmol/kg ip., daily for 8 months	OF: no diff. NOR: ↑ recognition index MWM: ↑ time in target quadrant	n.d.	↓ Aβ plaque load, ↓ Iba-1 + microglia, ↑ Doublecortin + neurons, ↑ synapse number in the cortex and CA1 area
[232]	Sprague–Dawley rats, intrahippocampal Aβ_25-35_	single dose of 0,05, 0,5, and 5 nmol intrahippocampal drug 30 min prior to Aβ_25-35_	MWM: ↓ escape latency (0,5 and 5 nmol), ↓ escape distance (0,5 and 5 nmol), ↑ time and distance in target quadrant	n.d.	n.d.
[188]	SAMP8 mice	100 or 500 μg/kg sc., daily for 4 months	active avoidance T-maze: ↑ memory retention NOR: no improvement	n.d.	↑ total CA1 pyramidal neuron number and density (100 μg/kg)
[207]	C57/BL6 mice, icv. Aβ_1-42_	25 nmol/kg sc., daily for 8 weeks	Y-maze: ↑ spontaneous alternation MWM: ↓ escape latency, ↑ time in target quadrant, and number of platform crosses	↑ GLP-1R, ↓ Ser396- and Ser202/199-p-Tau, but not total Tau in the hippocampus ↑ Ser473-p-Akt and Ser9-p-GSK-3β in the hippocampus	↓ Tau phosphorylation in the cortex
[220]	hAPP_Lon_/PS1_A246E_ mice hAPP_swe_/hPS1_dE9_ mice	100 or 500 μg/kg sc., daily for 3 months 500 μg/kg sc., daily for 5 months	MWM: no diff. NOR: no diff. active avoidance T-maze: no diff.	n.d.	no atrophy and no diff. in Aβ plaque load in cortex, hippocampus, and striatum (both models)
[208]	PLB4 mice	25 nmol/kg five times a week for 10 weeks	No difference in habituation activity	↑ serum DPP-4, IR mRNA ↓ Rbp4, Dpp4, Chrebp, Srebp1c mRNA	n.d.
[206]	Kunning mice, icv. STZ	300 μg/kg sc., daily for 30 days	MWM: ↓ escape latency, escape distance, ↑ time in target quadrant, and hidden platform crossings	↓ p-NF-M/H, ↑ glycated-NF-M/H ↓ Ser199/202-, Ser396/404-, Ser214-, Thr212-, and Thr231-p-Tau ↑ microtubule binding of Tau ↑ p-ERK1, ↓ p-JNK1/2	↓ p-NF-M/H, ↑ glycated-NF-M in cortex ↓ Ser199/202-p-Tau in cortex and CA1 ↓ degenerated neurons in CA1, CA4 and cortex
[205]	APP_swe_/PS1_dE9_ mice	0.2 mg/kg sc., daily for 2 months	n.d.	No changes on plasma leptin ↓ Thr231-p-Tau, caspase-3 in the hippocampus	↓ Aβ plaque load in the CA1 region ↓ GFAP+ astrocyte in cortex, no diff. in Iba-1+ microglia
[211]	3xTg-AD mice	300 μg/kg sc., daily for 8 weeks	MWM: ↓ escape latency, escape distance, ↑ time in target quadrant, and number of platform crossings	↓ Thr231-, Ser214-, Ser396-, S199/202-p-Tau, but not total Tau ↓ NF-H and NF-M ↑ p-ERK1/2, ↓ p-JNK1/2	↓ degenerated neurons in CA3, CA4 and cortex
[195]	Male Swiss mice, icv. AβOs	25 nmol/kg ip., daily for 7 days	NOR, OLM: ↑ novel/displaced object exploration time Contextual fear conditioning: ↑ freezing behavior	↑ PKA in the hippocampus ↑ IRα mRNA in the hippocampus	n.d.
	Non-human primates (macaques), icv. AβOs	0.006 mg/kg for 7 days, then 0.012 mg/kg for 24 days	n.d.	↓ Ser396-p-Tau (frontal cortex)	↓ p-Tau in the frontal cortex ↑ IRα and IRβ in the cortex, and IRα in the hippocampus
[229]	APP_swe_/PS1_dE9_ mice	25 nmol/kg ip., daily for 7 and 37 days	n.d.	n.d.	↑ cell proliferation, Doublecortin+ neurons (acute and chronic), and NeuN+ neurons (chronic)
[217]	APP_swe_/PS1_dE9_ mice	25 nmol/kg or 2,5 nmol/kg ip., daily for 10 weeks	OF: no difference NOR: ↑ recognition index (25 nmol/kg only)	n.d.	↓ Aβ plaque load, Iba-1+ microglia in the cortex (both doses) ↑ synaptic density in the hippocampus and cortex (both doses)
[224]	hTauP301L mice	500 μg/kg sc., daily for 22 weeks	n.d.	n.d.	↓ total neuronal p-Tau
[218]	APP_swe_/PS1_dE9_ mice	25 nmol/kg ip. daily, or sc. implant	n.d.	n.d.	↓ Aβ plaque load in the cortex, CA1, and dentate gyrus ↓ Iba-1 + microglia and GFAP + astrocyte in the hippocampus
[209]	3xTg-AD mice	0.2 mg/kg sc., daily for 28 days	OF: no difference Y-maze: no difference MWM: no difference	↓ Aβ_1-42_, CRP, pyruvate, reactive carbonyl groups and nitrite level, ↑ G6PDH activity	n.d.
[214]	APP/PS1 mice	25 nmol/kg ip., daily for three months	MWM: ↑ number of platform crossings Y-maze: ↑ frequency in the novel arm	↓ Aβ_1-42_, GSK-3β ↑ SYP, GLP-1R, PI3K and Akt	↓ Aβ plaque load, GSK-3β and ↑ neurons, SYP, PSD95, GLP-1R, p-PI3K and Akt immunoreactivity in the hippocampus
[154]	5xFAD mice	25 nmol/kg sc., daily for 8 weeks	n.d.	↑ cAMP, p-PKA, ATP production, and ↓ ROS	↑ neurons in the cortex
[210]	App^NL-G-F^ mice	200 μg/kg sc., daily for 4 and 20 weeks	Y-maze: ↑ spontaneous alternation	↑ PKA activity in the cortex (4 weeks)	↓ Aβ_1-42_ positive area and reactive astrocytes, ↑ Ser-p-AQP4 in the cortex (4 weeks)
[219]	3xTg-AD mice	0.2 mg/kg sc., daily for four months	n.d.	↓ NF-κB, JNK no diff. in Ser396-p-Tau, GSK-3β, PP2A	↓ Aβ plaque load in the CA1 region and GFAP+ astrocytes in the amygdala

The table presents statistically significant results (no trends of increase/decrease), the arrows indicating the direction of change/difference following drug administration compared to placebo-treated animals with AD. ADAM10: disintegrin and metalloproteinase domain-containing protein 10; Akt: RAC-alpha/gamma serine/threonine–protein kinase; APP: amyloid precursor protein; AQP4: aquaporin-4; ATP: adenosine triphosphate; Aβ: amyloid-β; AβO: amyloid-β oligomer; BACE1: β-site amyloid precursor protein cleaving enzyme; BDNF: brain-derived neurotrophic factor; cAMP: cyclic adenosine monophosphate; CA1/CA3/CA4: *Cornu Ammonis* 1/2/3; CRP: C-reactive protein; DPP-4: dipeptidyl peptidase-4; ERK1/2: extracellular signal-regulated kinase 1/2; GFAP: glial fibrillary acidic protein; GLP-1R: glucagon-like peptide-1 receptor; GSK-3β: glycogen synthase kinase-3; G6PDH: glucose-6-phosphate dehydrogenase; Iba-1: ionized calcium binding adaptor molecule 1; IDE: insulin-degrading enzyme; IGF-1R: insulin-like growth factor 1 receptor; IR: insulin receptor; IRS-1: insulin receptor substrate 1; JNK: c-Jun N-terminal kinase; MWM: Morris Water Maze; n.d.: no data; NF-M/H: medium/high molecular weight neurofilament; NF-κB: nuclear factor κB; NOD: Novel Object Discrimination Task; NOR: Novel Object Recognition test; OF: Open Field Test; OLM: object location memory test; PA: passive avoidance learning; PI3K: phosphoinositide 3-kinase; PKA: protein kinase A; PP2A: protein phosphatase 2A; PSD95: postsynaptic density protein 95; PSEN1: presenilin-1; ROS: reactive oxygen species; Ser: serine; STZ: streptozotocin; SYN/SYP: synaptophysin; Thr: threonine.

### 6.3. Lixisenatide

The published data on lixisenatide in animal models of AD are limited (Table 3). In two separate studies, Cai et al. tested the protective effect of intrahippocampally administered lixisenatide against Aβ-induced neurotoxicity [233,234]. Spatial memory preservation was confirmed by the Y-maze and the MWM. Reduced overactivation of GSK-3β in the hippocampus was documented, similar to exenatide and liraglutide [162,182,207]. Later, the same author found that chronic ip. administration of lixisenatide reversed the suppression of the PKA/CREB signaling pathway and reduced the phosphorylation of stress response element p38 MAPK, which is involved in Tau and IRS-1 phosphorylation [235]. These findings are consistent with observations made in vitro on primary hippocampal neurons: lixisenatide, when administered before Aβ_25-35_, enhanced cell viability and activation of Akt, and mitogen-activated extracellular signal-regulated kinase 1 and 2 (MEK1/2) [234]. Histological hallmarks, including Aβ plaque and p-Tau density, as well as microglial activation, showed promising trends following chronic treatment [217,235]. In addition, both studies documented improved LTP regardless of the different models applied [217,233].

### 6.4. Semaglutide and Other GLP-1RAs

Semaglutide is currently emerging as a promising multifaceted therapeutic agent with undoubtful neuroprotective effects [236]. There is evidence that it improved learning and memory capacities (assessed with NOR, Y-maze, MWM, and Barnes maze) and reduced Aβ plaque and NFT load in 3xTg-AD and APP/PS1 mice [237,238,239]. Zhang et al. were the first to show that the recovery of cognitive functions by GLP-1RA administration is associated with the restoration of oxytocin expression, completing our understanding of their neurotrophic role in AD [238]. Semaglutide also remediated glucose metabolism by regulating the GLUT4 expression and translocation after chronic administration [237]. The transition of microglia state from M1 to M2 was identified by Wang et al. under the influence of semaglutide [239]. This transition correlated with cortical and hippocampal decrease in IL-1β and TNF-α mRNA, while the expression of anti-inflammatory cytokines, IL-4 and IL-10, increased. Similar trends of microglia polarization and inflammatory cytokines were identified in the P301S model of tauopathy [240]. In this study additional neuroprotective and autophagy mediating mechanisms of GLP-1RA’s have been proposed, such as upregulation of angiotensin-converting enzyme 2/Angiotensin-(1-7)/Mas receptor, sirtuin 1/forkhead box protein O1 (SIRT1/FOXO1), and adenosine monophosphate-activated protein kinase (AMPK)/GSK-3β (Ser9) signaling. However, contradictory findings have also been reported. Administration of 25 nmol/kg semaglutide once daily did not alter the histological presentation of Aβ in the cortex and hippocampus of 5xFAD and APP/PS1 mice [241]. Furthermore, no relief of gliosis or improvement of cognitive performance was measurable after treatment. Boboc et al. found no compelling evidence in a series of applied behavioral tests [242]. Several studies are investigating the neuroprotective properties of semaglutide in Parkinson’s disease experimental models, concluding beneficial effects on locomotor activity, dopaminergic cell function, and microglia activation [243,244]. A recent systematic review collected existing data on the neuroprotective and cognitive effects of semaglutide [245], according to which it mitigated neuroinflammation and apoptosis in acute ischemic stroke [246], epilepsy [247], and multiple sclerosis [248].

Dulaglutide and albiglutide (withdrawn in 2017) have not been extensively studied in neurodegenerative disease experimental models. Dulaglutide was found to improve spatial learning (MWM) in rats with STZ-induced neuroinflammation. Dulaglutide also reduced Tau phosphorylation and restored PI3K/Akt/GSK-3β cascade activity after four weeks of treatment [249]. Impressive neuroprotective properties have been associated with NLY01 in two different transgenic models of AD. This long-acting pegylated exendin-4 analog exhibited beneficial changes in behavioral aspects (Y-maze and MWM) and neuroinflammation (astrocytosis and cytokines). Similar to exenatide, NLY01 increased BDNF and IDE expression in the hippocampus of 5xFAD and 3xTg-AD animals [184].

### 6.5. Dual GLP-1/GIP Receptor Agonists and Other Incretin-Based Multi-Agonists

Despite the considerable number of molecules currently under development, only tirzepatide, retatrutide, and the Hölscher peptides have been tested in experimental animal models of AD (Table 4).

Data concerning the disease-modifying properties of tirzepatide are conflicting. However, the drug has been shown to positively influence diabetes-associated central insulin resistance, neuroinflammation, and cognitive decline [250], but it failed to reach improvement in the APP/PS1 and 5xFAD AD models [241,251]. Beneficial changes in the expression of glucose metabolism-related transporter (GLUT1) and enzymes (hexokinase, G6PDH, phosphofructokinase) have been observed in the cortex but not in the hypothalamus of APP/PS1 mice [251].

The double agonist DA-JC1 provoked amelioration of histological presentations of AD and reduced gliosis to a greater extent compared to liraglutide [136]. Similarly, the administration of DA-CH3, DA4-JC, and DA5-CH led to a reduction in activated microglia and reactive astrocyte immunoreactivity, as well as a decrease in Aβ plaque and phosphorylated tau load (Table 4). Better synapse preservation could also be observed compared to untreated animals [135,252,253]. DA4-JC and DA5-CH both remediated synaptic plasticity in 3xTg-AD and APP/PS1 mice [253,254,255]. The reduced Bax/Bcl-2 ratio indicates diminished proapoptotic signaling by DA4-JC and DA5-CH in the icv. STZ-induced AD model [135,256]. The PINK1/Parkin interplay regulated the remediation of defective mitochondrial autophagy [257] and could establish the basis of improved learning and memory capacities assessed with MWM, Y-maze, and NOR [253] (Table 4). Moreover, DA-CH3 alleviated endoplasmic reticulum-specific stress and autophagy [252]. In the study conducted by Maskery et al., DA4-JC reduced the level of pro-inflammatory cytokines, IL-1β, and TNF-α and proved to be superior to liraglutide in all tested parameters [255]. Analogous to the previously presented GLP-1RAs, the positive regulation of the IRS-1 and the PI3K/Akt/GSK-3β signaling pathway by these experimental drugs was confirmed in multiple studies [252,254,256].

A GLP-1/GIP/glucagon receptor triagonist improved synaptic transmission and neuronal excitability in 3xTg-AD mice [258]. Retatrutide also rescued working and spatial memory, parallel with the activation of the cAMP/PKA/CREB pathway in 3xTg-AD mice [259]. In APP/PS1 animals, retatrutide (10 nmol/kg ip., two months) improved MWM performance; ameliorated cortical and hippocampal Aβ plaque load, inflammation, and oxidative stress; and increased BDNF levels [260]. The GLP-1 and glucagon receptor agonist (D-Ser2)-oxyntomodulin exhibited analogous beneficial effects to the previously presented multi-agonists. Cognitive impairment, Aβ pathology, LTP, and the PI3K/Akt/GSK-3β signaling cascade all demonstrated enhancement in the APP/PS1 transgenic mouse model [261].

**Table 4:** Studies assessing dual GLP-1/GIP receptor agonists in animal models of Alzheimer’s disease.

Drug and Study	Animal Model	Route of Administration and Dosage	Results		
			Behavior	Biochemistry	Histology
DA-JC1 [136]	APP_swe_/PS1_dE9_ mice	50 nmol/kg ip., daily for 4 weeks	n.d.	n.d.	↑ doublecortin+ neurons in the subventricular zone ↓ Aβ plaque load in the cortex, CA1, and dentate gyrus ↓ GFAP + astrocytes and Iba-1 + microglia in the hippocampus
DA-CH3 [252]	APP_swe_/PS1_dE9_ mice	25 nmol/kg ip., daily for 8 weeks	MWM: ↓ escape latency and distance moved, ↑ time in target quadrant reversal MWM: ↑ time in target quadrant	↑ PSD95 and SYP ↑ Thr308-p-Akt and Ser9-p-GSK-3β	↓ Aβ plaque load, ↓ GFAP + astrocytes, and Iba-1 + microglia in the cortex and hippocampus
DA4-JC [253]	3xTg-AD mice	10 nmol/kg ip., daily for 46 days	ORT: ↑ recognition index Y-maze: ↑ spontaneous alternation MWM and reversal MWM: ↑ time in target quadrant and platform crossings Conditional fear memory test: ↑ freezing behavior	↑ PSD95 and SYP ↑ PINK1/Parkin, ↓ P62 in the hippocampus	↑ number of synapses and dendritic spines ↑ volume and ↓ number of mitochondria in the hippocampus (TEM) ↓ Aβ- and tau-area in the hippocampus
DA4-JC [255]	APP_swe_/PS1_dE9_ mice	10 nmol/kg ip., daily for 6 weeks	MWM: ↑ time in target quadrant	↓ IL-1β and TNF-α	↓ Aβ plaque load, ↓ GFAP + astrocytes and Iba-1 + microglia in the cortex
DA4-JC [256]	Sprague-Dawley rats, icv. STZ	10 nmol/kg ip., daily for 2 weeks	Y-maze: ↑ spontaneous alternation MWM: ↓ escape latency, ↑ time in target quadrant	↓ Bax/Bcl-2 ratio ↑ p-Akt, ↓ p-IRS-1 in the cortex and hippocampus	↓ Ser396-p-tau, ↓ GFAP + astrocytes and Iba-1 + microglia in the cortex and hippocampus
DA5-CH [254]	APP_swe_/PS1_dE9_ mice	10 nmol/kg ip., daily for 4 weeks	Y-maze: ↑ spontaneous alternation MWM and reversal MWM: ↓ escape latency, ↑ time in target quadrant, and platform crossings	↑ p-PI3K, p-Akt, ↓ p-GSK-3β	↓ Aβ- and p-tau-immunoreactivity in the hippocampus
DA5-CH [135]	Sprague-Dawley rats, icv. STZ	10 nmol/kg ip., daily for 2 weeks	Y-maze: ↑ spontaneous alternation MWM: ↑ time in target quadrant and platform crossings	↓ Ser396-p-tau, ↓ Bax/Bcl-2 ratio, ↑ PSD95 and SYN, ↑ p-CREB in the hippocampus	↓ Ser396-p-tau, ↑ PSD95 and SYN in the hippocampus
Tirzepatide [251]	APP/PS1	10 nmol/kg ip., daily for 8 weeks	NOR: no difference in recognition index	↓ GLP-1R, GFAP, BACE1, ↑ GLUT1, HK, G6PDH, PFK mRNA in the cortex	↓ Aβ plaque number in the cortex, ↓ GFAP + astrocytes in the cortex and hippocampus
Tirzepatide [241]	5xFAD mice	10 nmol/kg sc., daily for 7 weeks	NOR: ↑ discrimination index (female mice) MWM: no difference	no difference in inflammation-related gene expression	no difference in Aβ plaque load, microglia and astrocyte activation

The table presents statistically significant results (no trends of increase/decrease), the arrows indicating the direction of change/difference following drug administration compared to placebo-treated animals with AD. Akt: RAC-alpha/gamma serine/threonine–protein kinase; Aβ: amyloid-β; BACE1: β-site amyloid precursor protein cleaving enzyme; BDNF: brain-derived neurotrophic factor; CA1: *Cornu Ammonis* 1; CREB: cAMP response element-binding protein; GFAP: glial fibrillary acidic protein; GLP-1R: glucagon-like peptide-1 receptor; GLUT1: glucose transporter type 1; GSK-3β: glycogen synthase kinase-3; G6PDH: glucose-6-phosphate dehydrogenase; HK: hexokinase; Iba-1: ionized calcium-binding adaptor molecule 1; IL-1β: interleukin 1β; IRS-1: insulin receptor substrate 1; MWM: Morris Water Maze; n.d.: no data; NOR: Novel Object Recognition test; ORT: Object Recognition Task; PFK: phosphofructokinase; PINK1: PTEN-induced kinase 1; PI3K: phosphoinositide 3-kinase; PKA: protein kinase A; PSD95: postsynaptic density protein 95; Ser: serine; STZ: streptozotocin; SYN/SYP: synaptophysin; TEM: transmission electron microscopy; Thr: threonine; TNF-α: tumor necrosis factor-α.

## 7. GLP-1RAs in Clinical Trials of AD

It has been observed in a large number of cases that the risk of all-cause dementia is lower in diabetic patients who use GLP-1RA compared to non-GLP-1RA users [262,263]. To elucidate whether this reduction is due to ameliorated diabetic and cardiovascular risk factors or AD-specific neuroprotection, further studies are needed. A large retrospective cohort also confirmed that the incidence of AD was significantly lower among exenatide users [264]. In a pilot study, exenatide did not modify neuropsychiatric disease scores or CSF and plasma biomarkers of early AD [265]. Similarly, no influence on MCI progression could be detected following 32 weeks of slow-release exenatide treatment [266]. Although there was no measurable positive effect of liraglutide on Aβ and cognition after 6 months, preservation of cerebral glucose transfer and metabolism of AD patients was detected compared to placebo [267,268]. Meta-analysis of five early studies with exenatide and liraglutide administration to AD and MCI diagnosed patients reports improved cognitive and memory functions following treatment [269]. However, sample sizes were small, treatment duration ranged from 12 weeks to 12 months, and some studies included patients with T2DM. The EVOKE and EVOKE+ are currently ongoing large-scale clinical trials that will provide data on oral semaglutide’s disease-modifying effect in early-stage symptomatic AD [270].

## 8. Conclusions

In conclusion, based on the above-presented preclinical data originating from transgenic and interventional animal models of AD, GLP-1RAs could be a promising therapeutic strategy in preventing and ameliorating AD-associated neurodegeneration. The fact that they have been approved for human therapeutic use for the last 20 years is compelling evidence of their safety and provides ample opportunity to evaluate their potential efficacy in AD-related states; however, clinical data have not provided convincing results so far. Nonetheless, the use of animal studies remains essential to enrich our pathomechanistic knowledge of this widely studied yet not fully comprehended disease.

## Figures and Tables

**Table 3:** Studies assessing lixisenatide in animal models of Alzheimer’s disease.

Study	Animal Model	Route of Administration and Dosage	Results		
			Behavior	Biochemistry	Histology
[217]	APP_swe_/PS1_dE9_ mice	1 or 10 nmol/kg ip., daily for 10 weeks	OF: no difference NOR: ↑ recognition index (both doses)	n.d.	↓ Aβ plaque load, Iba-1+ microglia in the cortex (both doses) ↑ synaptic density in the hippocampus and cortex (both doses)
[233]	Spargue-Dawley rats, intrahippocampal Aβ_25-35_	10 nmol intrahippocampal drug 15 min prior to Aβ_25-35_	MWM: ↓ escape latency, ↑ time in target quadrant	↑ Ser9-p-GSK-3β and ↓ Tyr216-p- GSK-3β in the hippocampus	n.d.
[234]	Spargue-Dawley rats, intrahippocampal Aβ_25-35_	10 nmol intrahippocampal drug 15 min prior to Aβ_25-35_	Y-maze: ↑ spontaneous alternation	n.d.	n.d.
[235]	APP/PS1/tau mice	10 nmol/kg ip., daily for 60 days	n.d.	↑ p-PKA, p-CREB ↓ p-p38 MAPK in the hippocampus	↓ Aβ plaque and p-Tau load, ↓ Iba-1+ microglia in the hippocampus

The table presents statistically significant results (no trends of increase/decrease), the arrows indicating the direction of change/difference following drug administration compared to placebo-treated animals with AD. Aβ: amyloid-β; CREB: cAMP response element-binding protein; GSK-3β: glycogen synthase kinase-3; Iba-1: ionized calcium-binding adaptor molecule 1; MWM: Morris Water Maze; n.d.: no data; NOR: Novel Object Recognition test; OF: Open Field Test; p38 MAPK: p38 mitogen-activated protein kinase; PKA: protein kinase A; Ser: serine; Tyr: tyrosine.

## Data Availability

No new data were created or analyzed in this study. Data sharing is not applicable to this article.

## Supplementary Materials

The following supporting information can be downloaded at: https://www.mdpi.com/article/10.3390/ph18050614/s1, Supplementary Table S1: Structural and pharmacokinetic comparison of the discussed glucagon-like peptide-1 receptor agonists (GLP-1RAs) and dual, triple incretin receptor agonists; Supplementary Figure S1: Structural comparison of the discussed glucagon-like peptide-1 receptor agonists (GLP-1RAs) and dual, triple incretin receptor agonists.

## Abbreviations

Ach—acetylcholine; AChE—acetylcholinesterase; AD—Alzheimer’s disease; ADAM10—disintegrin and metalloproteinase domain-containing protein 10; AGE—advanced glycation end-product; Akt—RAC serine/threonine–protein kinase; AKT1—RAC-alpha serine/threonine–protein kinase (gene), AKT3—RAC-gamma serine/threonine–protein kinase (gene); AMPK—adenosine monophosphate-activated protein kinase; apoE4—apolipoprotein E4; APP—amyloid-β precursor protein; AQP4—aquaporin-4; ARE—antioxidant response element; Aβ—amyloid-β; AβO—amyloid-β oligomer; BACE1—β-site amyloid precursor protein cleaving enzyme; BDNF—brain-derived neurotrophic factor; BMI—body mass index; CA1/CA3/CA4—*Cornu Ammonis* brain regions 1/3/4; CaMKII-α—Ca^2+^/calmodulin-simulated protein kinase II alpha; cAMP—cyclic adenosine monophosphate; ChAT—choline acetyltransferase; CLHLS—Chinese Longitudinal Healthy Longevity Survey; CNS—central nervous system; COX—cyclooxygenase; CREB—cAMP response element-binding protein; CRP—C reactive protein; CTNNB1—catenin beta 1 protein (gene); CSF—cerebrospinal fluid; DMT—disease-modifying therapy; DPP-4—dipeptidyl peptidase-4; Epac—exchange protein of cAMP; ERK—extracellular signal-regulated kinase; GFAP—glial fibrillary acidic protein; GIP—gastric inhibitory polypeptide; GlcNAc—N-acetylglucosamine; GLP-1—glucagon-like peptide-1; GLP-1R—glucagon-like peptide-1 receptor; GLP-1RA—glucagon-like peptide-1 receptor agonist; GLUT1/GLUT3/GLUT4—glucose transporter type 1/3/4; GnT-III—N-acetylglucosaminyltransferase III; GSK-3β—glycogen synthase kinase-3 beta; G6PDH—glucose-6-phosphate dehydrogenase; HbA1c—glycated hemoglobin type A; HK—hexokinase; HFD—high-fat diet; HOMA-IR—homeostatic model assessment-insulin resistance classification system; IAPP—islet amyloid polypeptide; Iba-1—ionized calcium-binding adaptor molecule 1; IDE—insulin-degrading enzyme; IFN—interferon; IGF-1—insulin-like growth factor 1; IGF-1R—insulin-like growth factor 1 receptor; IL—interleukin; INSR—insulin receptor (gene); IR—insulin receptor; IR-A/IR-B—insulin receptor isoform A/B; IRS—insulin receptor substrate; JAK—Janus kinase; JNK—c-Jun N-terminal kinase; Keap—Kelch-like ECH-associated protein 1; LDH—lactate dehydrogenase; LPS—lipopolysaccharide; LTP—long-term potentiation; MAPK—mitogen-activated protein kinase; MCI—mild cognitive impairment; MEK1/2—mitogen-activated protein kinase; MMSE—Mini-Mental State Examination; mTOR—mammalian target of rapamycin; MWM—Morris water maze; n.d.—no data; NADPH—nicotinamide adenine dinucleotide phosphate; NeuN—hexaribonucleotid binding protein 3; NF-kB—nuclear factor kappa B; NF-M/H—medium/high molecular weight neurofilament; NFT—neurofibrillary tangle; NHP—non-human primates; NLRP2/NLRP3—nucleotide-binding oligomerization domain, leucine-rich repeat and pyrin domain containing 2/3; NMDA—N-methyl-D-aspartate; NOD—new object discrimination; NOR—Novel Object Recognition; Nrf2—nuclear erythroid-2-like factor-2; ODT—object displacement task; OLM—object location memory; ORT—object recognition task; PAL—passive avoidance learning; PFK—phosphofructokinase; PGE2—prostaglandin E2; PINK1—PTEN-induced kinase 1; PI3K—phosphoinositide 3-kinase; PKA—protein kinase A; PP2A—protein phosphatase 2 enzyme; PPARα/γ—peroxisome proliferator-activated receptor α/γ; PPG—preproglucagon; PSD95—postsynaptic density protein 95; PSEN1—presenilin 1 protein; PTP1B—protein-tyrosine phosphatase 1B enzyme; RAGE—receptors for advanced glycation end-product; RNS—reactive nitrogen species; ROS—reactive oxygen species; SAMP8—senescence accelerated mouse-prone 8; Ser—serine; SLC2A2—solute carrier family 2 member 2 protein coding gene; SOD—superoxide dismutase; STAT3—signal transducers and activators of transcription 3; STZ—streptozotocin; SYN/SYP—synaptophysin; T2DM—type two diabetes mellitus; TCF/LEF—T-cell factor/lymphoid enhancer-binding factor; TCPTP—T-cell protein tyrosine phosphatase; TEM—transmission electron microscopy; TGFβ—transforming growth factor; Thr—threonine; TNF-α—tumor necrosis factor alpha; Tyr—tyrosine
