# Supplementary material for: Antidiabetic GLP-1 Receptor Agonists Have Neuroprotective Properties in Experimental Animal Models of Alzheimer’s Disease
Source: Pharmaceuticals (Basel). 2025 Apr 23;18(5):614. doi: 10.3390/ph18050614 (PMC12114801; doi:10.3390/ph18050614)

Supplementary Table S1. Structural and pharmacokinetic comparison of the discussed glucagon-like peptide-1 receptor agonists (GLP-1RAs) and dual, triple incretin receptor agonists

Compound		Exenatide	Liraglutide	Lixisenatide	Semaglutide	Dulaglutide	Albiglutide	Tirzepatide	Retatrutide
**Commercial names, product type and indication**		**Byetta** immediate-release product (solution) for treatment of T2DM, for EU and US markets; **Bydureon** prolonged-release product (solution) for treatment of T2DM, for EU and US markets;	**Victoza** immediate-release product (solution) for treatment of T2DM, for EU and US markets; **Saxenda** immediate release product (solution) for body weight management, for EU and US markets;	**Lyxumia** immediate-release product (solution) for treatment of T2DM, for EU market; **Adlyxin** immediate release (solution) product for treatment of DM for US market, withdrawn in 2023;	**Ozenpic** immediate-release product (solution) for treatment of T2DM, for EU and US markets; **Rybelsus** immediate-release product (tablet) for treatment of T2DM, for EU and US markets; **Wegovy** immediate-release product (solution) for body weight control, for EU and US markets;	**Trulicity** immediate-release product (solution) for treatment of T2DM, for EU and US markets;	**Eperzan** immediate-release product (solution) for treatment of T2DM, for EU market; **Tanzeum** immediate release product (solution) for treatment of T2DM, for US market, withdrawn in 2018;	**Mounjaro** immediate-release product (solution) for treatment of T2DM and body weight management, for EU and US markets; **Zepbound** immediate release product (solution) for body weight management, for US market;	Currently ongoing phase 3 clinical trial for treatment of T2DM and body weight management;
**Structural features**	**Structure**	synthetic GLP-1 analogue consisting of 39 amino acids with partial sequence homology to the naturally occurring human GLP-1	analogue of human GLP-1 in which the lysine residue at position 27 is replaced by arginine and a hexadecanoyl group attached to the	synthetic GLP-1 analogue containing 44 amino acids, which is amidated at the C terminal amino acid; resistant to enzymatic cleavage by DPP-4	synthetic GLP-1 analogue with following modifications<br>- position 8: alanine to 2-aminoisobutyric acid)<br>- position 34: lysine to an arginine)	two identical synthetic GLP-1 analogues linked to IgG4 domain; homodimer structure with two identical polypeptide	two identical synthetic GLP-1 analogues linked to human albumin; glycine substituted for the naturally-occurring alanine at	synthetic GLP-1 analogue consisting of 39 amino acids, containing<br>- two non-coded amino acids (Aib) in	synthetic GLP-1 analogue containing<br>- two non-coded amino acids (Aib);<br>- a large hydrophilic spacer

			remaining lysine via a glutamic acid spacer		- a large hydrophilic spacer between the lysine in position 26 and the gamma glutamate<br>- C18 fatty di-acid with a terminal acidic group	chains linked with disulfide bonds	position 8 to confer resistance to DPP-4	positions 2 and 13,<br>- a C-terminal amide<br>- Lys residue at position 20 attached to 1,20-eicosane dioic acid via a linker which consists of a γ-Glu and two 8-amino-3,6-dioxaoctanoic acids	attached to the lysine in position 17
	**Condensed IUPAC name with amino acid sequence**	H-His-Gly-Glu-Gly-Thr-Phe-Thr-Ser-Asp-Leu-Ser-Lys-Gln-Met-Glu-Glu-Glu-Ala-ValArg-Leu-Phe-Ile-Glu-Trp-Leu-Lys-Asn-Gly-Gly-Pro-Ser-Ser-Gly-	H-His-Ala-Glu-Gly-Thr-Phe-Thr-Ser-Asp-Val-Ser-Ser-Tyr-Leu-Glu-Gly-Gln-Ala-Ala-Lys(1)-Glu-Phe-Ile-Ala-Trp-Leu-Val-Arg-Gly-Arg-Gly-OH;	H-His-Gly-Glu-Gly-Thr-Phe-Thr-Ser-Asp-Leu-Ser-Lys-Gln-Met-Glu-Glu-Glu-Ala-Val-Arg-Leu-Phe-Ile-Glu-Trp-Leu-Lys-Asn-Gly-Gly-Pro-Ser-Ser-Gly-Ala-	H-His-Aib-Glu-Gly-Thr-Phe-Thr-Ser-Asp-Val-Ser-Ser-Tyr-Leu-Glu-Gly-Gln-Ala-Ala-Lys(C18-diacid-gamma-Glu-ADO-ADO)-Glu-Phe-Ile-Ala-Trp-Leu-Val-	The GLP-1 analogue moiety of the Dulaglutide molecule: H-His-Gly-Glu-Gly-Thr-Phe-Thr-Ser-Asp-Val-Ser-Ser-Tyr-	The GLP-1 analogue moiety of the Albiglutide: H-His-Gly-Glu-Gly-Thr-Phe-Thr-Ser-Asp-Val-Ser-Ser-Tyr-Leu-Glu-Gly-Gln-Ala-Ala-Lys-Glu-Phe-Ile-	H-Tyr-Aib-Glu-Gly-Thr-Phe-Thr-Ser-Asp-Tyr-Ser-Ile-Aib-Leu-Asp-Lys-Ile-Ala-Gln-Lys(Eicosanedioyl-isoGlu-ADO-ADO)-Ala-Phe-Val-Gln-Trp-Leu-	Tyr-Aib-Gln-Gly-Thr-Phe-Thr-Ser-Asp-Tyr-Ser-Ile-α MeLeu-Leu-Asp-Lys-Lys(ADO-γ-Glu-Eicosanedioic acid)-Ala-Gln-Aib-Ala-Phe-Ile-Glu-

	Ala-Pro-Pro-Pro-Ser-NH_2	palmitoyl-Glu(1)-OH	Pro-Pro-Ser-Lys-Lys-Lys-Lys-Lys-Lys-NH2	Arg-Gly-Arg-Gly-OH	Leu-Glu-Glu-Gln-Ala-Ala-Lys-Glu-Phe-Ile-Ala-Trp-Leu-Val-Lys-Gly-Gly-Gly-OH	Ala-Trp-Leu-Val-Lys-Gly-Arg-NH2	Ile-Ala-Gly-Gly-Pro-Ser-Ser-Gly-Ala-Pro-Pro-Pro-Ser-NH_2	Tyr-Leu-Leu-Glu-Gly-Gly-Pro-Ser-Ser-Gly-Ala-Pro-Pro-Pro-Ser-NH2
**Administration route**	Sc.	Sc.	Sc.	**Ozempic/Wegovy**: sc.<br>**Rybelsus**: oral	Sc.	Sc.	Sc.	Sc.
**Posology**	**Byetta**: twice daily with increasing doses from 5 to 10 μg;<br>**Bydureon**: 2 mg once a week	**Vidoza**: once daily, with increasing doses from 1.2 to 1.8 mg;<br>**Saxenda**: daily 3 mg	**Lyxumia/Adlyxin**: once daily with increasing doses from 10 to 20 μg	**Ozempic**: once weekly with increasing doses from 0.25 to 1 mg;<br>**Rybelsus**: once daily with increasing doses from 3 to 14 mg;<br>**Wegovy**: once weekly with increasing doses from 0.25 to 2.4 mg	**Trulicity**: once weekly with increasing doses from 0.75 to 4.5 mg	**Eperzan/ Tanzeum**: once weekly with increasing doses from 30 to 50 mg	**Mounjaro/ Zepbound**: once weekly with increasing doses from 2.5 to 15 mg	Not available
**Peak plasma concentration (t_{max})**	2.1 h	~12 h	1-3.5 h	60 h after sc. injection;<br>1.5 h after oral administration	48 h	3-5 days	~24 h (ranging from 8 to 72 h)	12-72.2 h
**Half-life**	2.4 h	~15 h	~3 h	145-148 h	102-107 h	3.6-6.8 days	~5 days	134-165 h
**Apparent clearance**	9.1 L/h	0.6-1.2 L/h	35 L/h	0.05 L/h	0.1 h	0.067 L/h	0.056 L/h	0.0267-0.0434 L/h

ADO: 8-amino-3,6-dioxaoctanoic acid; Aib: 2-aminoisobutyric acid; DPP-4: dipeptidyl peptidase-4; T2DM: type 2 diabetes mellitus. **Sources**: EMA Assessment Report of the Pharmaceutical Products, FDA Product Information Documentation and PubChem Database

Figure S1. Structural comparison of the discussed glucagon-like peptide-1 receptor agonists (GLP-1RAs) and dual, triple incretin receptor agonists

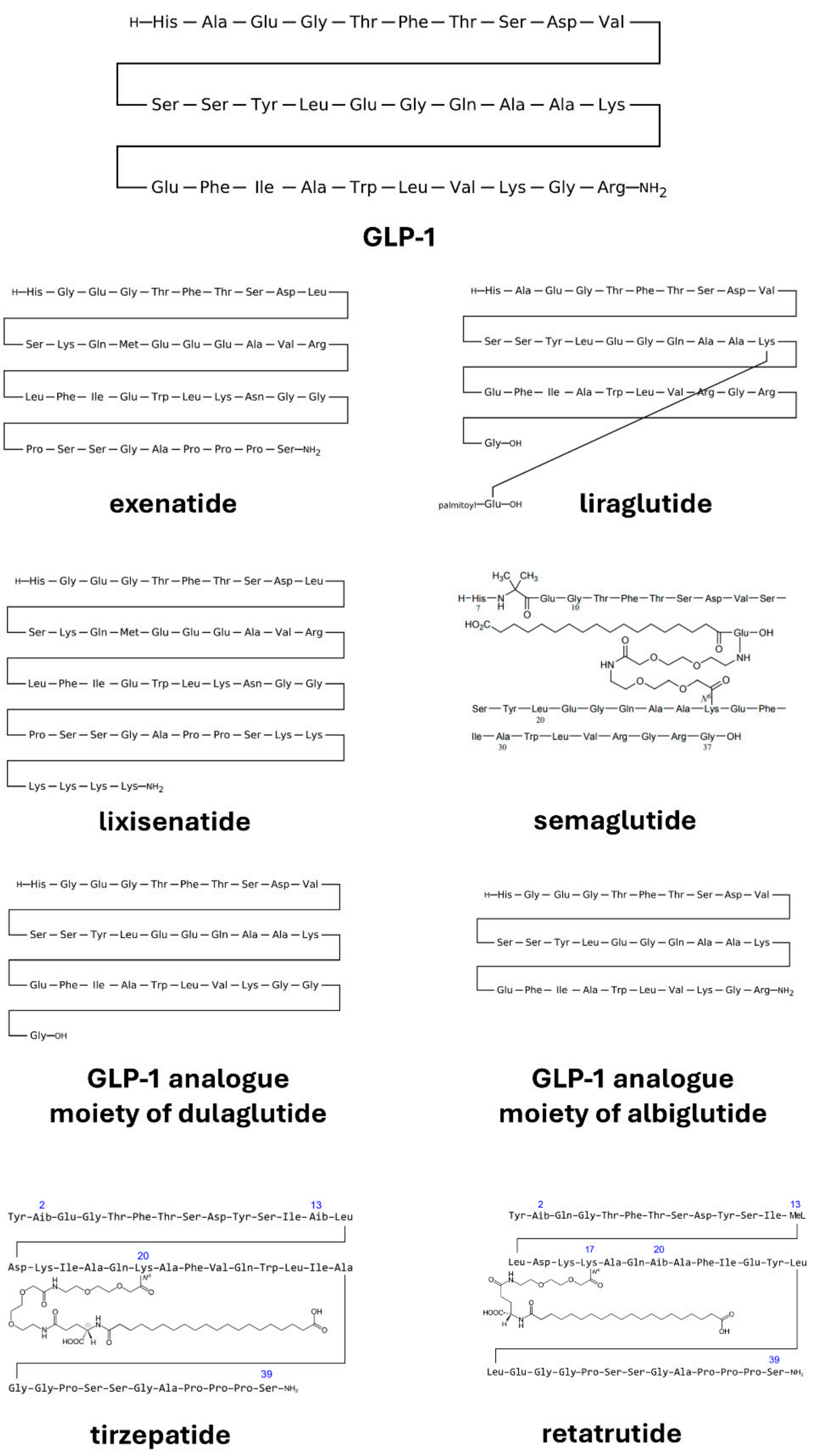

Supplement: Supplementary file 1 [file pharmaceuticals-18-00614-s001.zip › pharmaceuticals-3515999-supplementary.pdf]
